# RNA is required for the integrity of multiple nuclear and cytoplasmic membrane‐less RNP granules

**DOI:** 10.15252/embj.2021110137

**Published:** 2022-03-31

**Authors:** Carolyn J Decker, James M Burke, Patrick K Mulvaney, Roy Parker

**Affiliations:** ^1^ Department of Biochemistry University of Colorado Boulder Boulder CO USA; ^2^ Howard Hughes Medical Institute University of Colorado Boulder Boulder CO USA; ^3^ BioFrontiers Institute University of Colorado Boulder Boulder CO USA

**Keywords:** condensates, membrane‐less organelles, RNA granules, RNase L, RNP granules, Organelles, RNA Biology

## Abstract

Numerous membrane‐less organelles, composed of a combination of RNA and proteins, are observed in the nucleus and cytoplasm of eukaryotic cells. These RNP granules include stress granules (SGs), processing bodies (PBs), Cajal bodies, and nuclear speckles. An unresolved question is how frequently RNA molecules are required for the integrity of RNP granules in either the nucleus or cytosol. To address this issue, we degraded intracellular RNA in either the cytosol or the nucleus by the activation of RNase L and examined the impact of RNA loss on several RNP granules. We find the majority of RNP granules, including SGs, Cajal bodies, nuclear speckles, and the nucleolus, are altered by the degradation of their RNA components. In contrast, PBs and super‐enhancer complexes were largely not affected by RNA degradation in their respective compartments. RNA degradation overall led to the apparent dissolution of some membrane‐less organelles, whereas others reorganized into structures with altered morphology. These findings highlight a critical and widespread role of RNA in the organization of several RNP granules.

## Graphical abstract

Targeted degradation of nuclear or cytoplasmic RNA uncovers differential roles for RNA in maintaining specific RNP granules.

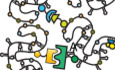

## Introduction

Eukaryotic cells contain many different compartments in the cytosol and nucleus that are not bound by membranes (Spector, 2006; Gomes & Shorter, 2019). Many of these membrane‐less organelles are large assemblies of RNA and protein, referred to as ribonucleoprotein (RNP) granules. RNP granules found in the cytoplasm include processing bodies or P‐bodies (PBs) (Jain & Parker, 2013), stress granules (SGs) (Anderson & Kedersha, 2006), U‐bodies (Liu & Gall, 2007), IMP granules (Jønson *et al*, 2007), FMRP granules (Mazroui *et al*, 2002), neuronal granules (Kiebler and Bassell, 2006), and germinal granules (Voronina *et al*, 2011). Examples of nuclear RNP granules include the nucleolus, nuclear speckles, paraspeckles, Cajal bodies, and nuclear SGs (Biamonti, 2004; Mao *et al*, 2011a). Understanding how these non‐membrane‐bound compartments or organelles assemble and maintain their integrity will provide fundamental insight on the organization of eukaryotic cells.

The role of RNA in organizing these compartments in the cell is an important question. There is evidence that RNA is required for the formation of some of these organelles. Several nuclear bodies are assembled on specific nascent transcripts which act as scaffolds to nucleate their assembly. For example, the long non‐coding RNA (lncRNA), NEAT1_2, plays a core architectural role in the assembly and organization of paraspeckles (Clemson *et al*, 2009; Sasaki *et al*, 2009). The transcription of precursor rRNA plays a critical role in the assembly of nucleoli. At the end of mitosis, nucleoli form around the sites of transcription of pre‐rRNA and nucleolar assembly also requires 45S rRNA produced prior to mitosis (Hernandez‐Verdun, 2011). RNAs are also critical for the assembly of RNA condensates in the cytoplasm. Formation of SGs and PBs requires non‐translating mRNAs (Kedersha *et al*, 1999; Teixeira *et al*, 2005; Buchan *et al*, 2008; Buchan & Parker, 2009).

Whether RNA plays an essential role in the formation of other RNP bodies is less clear. Cajal bodies, which are involved in the biogenesis and recycling of several different classes of small nuclear RNPs (snRNPs), form at genomic sites where individual Cajal body proteins are concentrated due to local transcription of snRNAs (Machyna *et al*, 2013; Sawyer *et al*, 2016), or being artificially tethered (Kaiser *et al*, 2008). Cajal body proteins are therefore able to nucleate the assembly of Cajal bodies. The role of RNA in the assembly of nuclear speckles is also unclear. A non‐coding RNA called MALAT1 is enriched in nuclear speckles, but it is not required for nuclear speckles to form (Hutchinson *et al*, 2007). However, poly(A)+ RNA is present in nuclear speckles under conditions where MALAT1 RNA localization to speckles is defective (Miyagawa *et al*, 2012), so whether other RNA species are involved in organizing nuclear speckles remains to be determined. Whether RNA is required for the stable maintenance of membrane‐less organelles once they are formed is also an unresolved issue.

One way that RNAs could promote RNP body assembly is by being a scaffold for RNA‐binding proteins which then bind to additional proteins either through homotypic or heterotypic protein–protein interactions and/or additional RNAs to form higher order assemblies (Fox *et al*, 2018). In addition, intermolecular RNA–RNA interactions can directly promote the assembly of RNP granules (Van Treek *et al*, 2018). Thus, RNP granule formation can be thought of as the summation of RNA–RNA, RNA–protein, and protein–protein interactions (Van Treek and Parker, 2018). The contribution of these three classes of interactions to the formation and stable maintenance of RNP assemblies may differ between different RNP granules or for the same type of RNP granule under different conditions.

We recently reported that SGs and PBs are differentially sensitive to the loss of RNA in the cytoplasm (Burke *et al*, 2020) suggesting that the role of RNA–RNA or RNA–protein interactions on their structural integrity differs. The antiviral endoribonuclease, ribonuclease L (RNase L), is activated in response to dsRNA which leads to widespread degradation of mRNAs in the cytoplasm (Burke *et al*, 2019; Rath *et al*, 2019). RNase L activity limits the formation of SGs and can disassemble preformed SGs (Burke *et al*, 2020). In contrast, RNase L activity does not dramatically affect the size of PBs (Burke *et al*, 2020). The differential response of SGs and PBs to loss of RNA in the cytoplasm suggests that SGs are strongly dependent on RNA–RNA and/or RNA–protein interactions, whereas PBs maybe more dependent on protein–protein interactions. Alternatively, RNAs within PBs may be stably associated and protected from RNase L in comparison to RNA associated with SGs.

To better understand the role of RNA in the organization of cellular compartments, we have examined whether a variety of RNP granules in the nucleus and the cytoplasm are dependent on the presence of RNA for their integrity. We quantified the effect of RNase L activation in the cytoplasm on the number and volume of PBs. We also targeted RNase L to the nucleus to induce the degradation of nuclear RNA and determine if nuclear bodies maintained their structure or were disassembled. We find that RNAs within nuclear RNP granules are susceptible to RNase L degradation and that granules disassemble, or form assemblies with altered morphology, in response to the loss of their resident RNAs. Taken together, our findings reveal that RNA plays an important role in organizing multiple cellular compartments.

## Results

### P‐body number and size are unaffected by RNase L activity

**Figure 1:**
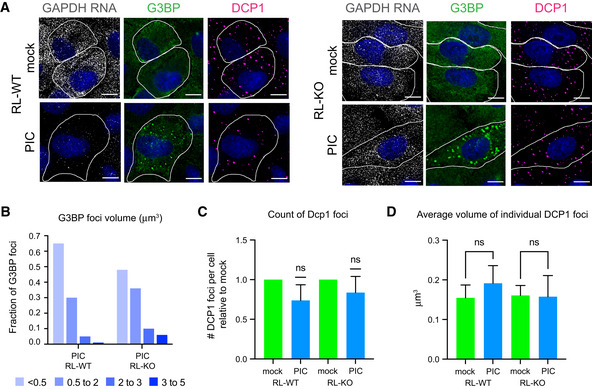
The number and size of P‐bodies is unaffected in cells with active RNase L GAPDH smFISH and IF analysis using anti‐G3BP antibody (G3BP) and anti‐Dcp1b antibody (DCP1) in A549 cells (RL‐WT) or A549 RL‐KO cells either mock transfected (mock) or transfected with poly(I:C) (PIC) for 5 h. Scale bar 10 microns.Graph of the fraction of G3BP foci with different volumes in PIC‐treated RL‐WT and RL‐KO cells.Number of DCP1 foci per cell in PIC‐treated RL‐WT and RL‐KO cells relative to the number in mock‐treated cells. Wilcoxon Signed Rank test, ns, non‐significant.Average volume of individual Dcp1 foci in mock‐ and PIC‐treated RL‐WT and RL‐KO cells. One‐way ANOVA with Sidak’s multiple comparisons test, ns, non‐significant. Data information: (C, D) Bar graphs show mean + SD for *N* = 4 independent experiments. Source data are available online for this figure.

RNase L activity has been reported to inhibit the assembly of SGs but has less effect on PBs (Burke *et al*, 2019, 2020). Similarly, we observed that SGs assembled in RNase L knockout (RL‐KO) A549 cells in response to poly(I:C), a viral dsRNA mimic that activates the RNase L pathway, but their assembly was prevented in wild‐type A549 cells with active RNase L, as judged by depletion of a cytoplasmic RNA (GAPDH RNA) (Fig 1A). In WT cells, only small G3BP foci assembled (Fig 1A and B), which are referred to as RNase L‐dependent bodies (RLBs) (Burke *et al*, 2019). RLBs are distinct from SGs having a different protein and RNA composition and forming independently of protein kinase R in response to dsRNA (Burke *et al*, 2020).

In contrast, we did not observe any effect of RNase L activation on PBs. In poly(I:C)‐treated cells that activated RNase L, the number of PBs per cell or the average size of PBs did not change significantly in response to poly(I:C) in the RL‐WT cells (Fig 1C and D) (Burke *et al*, 2020).

**Figure EV1:**
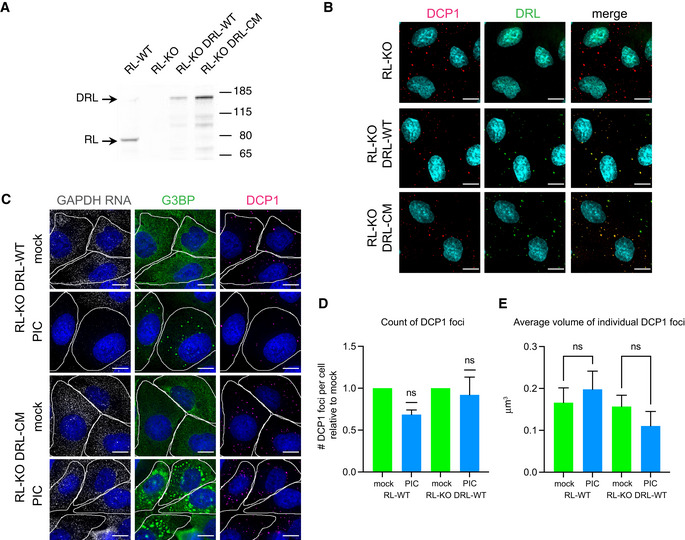
Targeting RNase L to P‐bodies did not increase the effect of RNase L activation on P‐body number or volume Full‐length Dcp1 RNase L (DRL) fusion proteins are expressed. Western analysis using anti‐RNase L antibody of whole cell lysates from A549 (RL‐WT) cells, A549 RNase L knock out cells (RL‐KO), and RL‐KO cells transduced with lentiviral vectors containing either wild‐type RNase L fused to DCP1a (RL‐KO DRL‐WT) or catalytic mutant RNase L‐R667A fused to DCP1a (RL‐KO DRL‐CM). Arrows on left indicate migration of the endogenous RNase L (RL) and the DCP1 RNase L fusion proteins (DRL).DRL fusion proteins co‐localize with P‐bodies. IF analysis using anti‐DCP1b antibody to detect P‐bodies (DCP1) or anti‐Flag antibody to detect Flag‐tagged DCP1a‐RNase L fusion proteins. Scale bar 10 microns.DRL‐WT fusion protein is active. GAPDH smFISH and IF analysis using anti‐G3BP antibody (G3BP) and anti‐Dcp1b antibody (DCP1) in A549 RL‐KO cells with DRL‐WT or DRL‐CM fusion proteins either mock transfected (mock) or transfected with poly(I:C) (PIC) for 5 h. Scale bar 10 microns.Number of DCP1 foci per cell in mock‐ and PIC‐treated RL‐WT and RL‐KO cells expressing DRL‐WT relative to the number in mock‐treated cells. Wilcoxon Signed Rank test, ns, non‐significantAverage volume of individual DCP1 foci in mock‐ and PIC‐treated cells. One‐way ANOVA with Sidak’s multiple comparisons test. Data information: (D, E) Bar graphs show mean + SD for *N* = 4 independent experiments. ns, non‐significant. Source data are available online for this figure.

One possibility for the differential effect of RNase L on PBs as compared to SGs is that RNase L is ineffective at accessing PBs. To test whether targeting RNase L to PBs would affect their integrity, we fused RNase L to a PB protein, Dcp1 (Ingelfinger *et al*, 2002) (Fig EV1A and B). Activation of the Dcp1‐RNase L fusion protein by PIC treatment caused the degradation of cytoplasmic RNA (Fig EV1C) but did not have a significant effect on the number or size of PBs (Fig EV1C–E). These results argue that the lack of an effect of activating RNase L on PBs is not due to RNase L being unable to access PBs. Although we cannot rule out that residual RNA species are responsible for maintaining PBs when cytoplasmic RNA is degraded, their persistence suggests that protein–protein interactions are sufficient to maintain their structure in the cell.

**Figure EV2:**
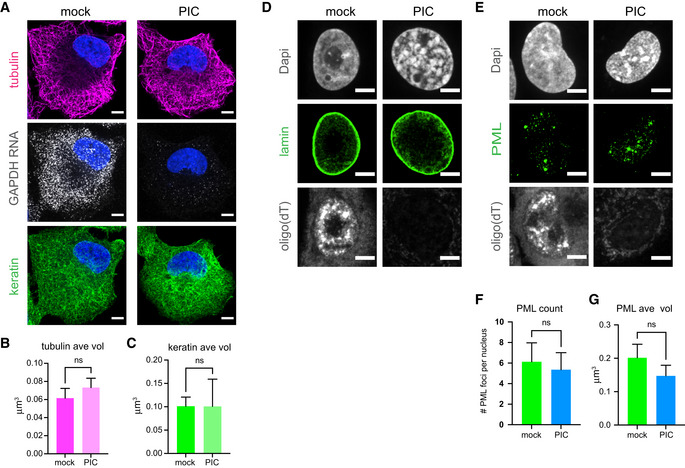
Degradation of RNA in the cytoplasm does not alter microtubule or intermediate filament networks and nuclear RNA degradation does not alter the integrity of the nuclear lamin or PML bodies AGAPDH smFISH and IF analysis of microtubules (anti‐alpha tubulin antibody) or intermediate filaments (anti‐pan keratin antibody) in A549 cells mock treated or treated with poly(I:C) for 5 h.BGraph of average volume of tubulin structures in mock‐ and PIC‐treated cells with reduced GAPDH RNA.CGraph of average volume of keratin structures in mock‐ and PIC‐treated cells with reduced GAPDH RNA.D, EA549 cells expressing nuclear‐localized wild‐type RNase L mock or poly(I:C) treated for 5 h. (D) Oligo(dT) FISH and IF analysis of nuclear lamin (anti‐lamin A antibody). (E) Oligo(dT) FISH and IF analysis of PML bodies (anti‐PML antibody).FGraph of the number of PML foci in mock‐ and PIC‐treated cells with reduced oligo(dT) signal.GGraph of average volume of PML foci in mock‐ and PIC‐treated cells with reduced oligo(dT) signal. Data information: All images scale bar 5 micron. (B, C) Mean and SEM of four experiments with at least 8 cells per condition per experiment analyzed. (F, G) Mean and SEM of three experiments with at least 10 cells per condition per experiment analyzed. All graphs unpaired two‐tailed *t*‐test, ns, non‐significant. Source data are available online for this figure.

RNase L activation did not lead to apparent changes in either the microtubule or intermediate filament networks (Fig EV2A and B) consistent with degradation of RNA in the cytoplasm specifically affecting RNA‐dependent assemblies.

### Method to degrade nuclear RNA in an inducible manner

The differential effect of loss of RNA on RNP granules in the cytoplasm led us to test whether RNP granules in the nucleus require RNA to be maintained. We reasoned that we could induce the degradation of nuclear RNA by targeting RNase L to the nucleus and activating its activity through the dsRNA innate immune response.

**Figure 2:**
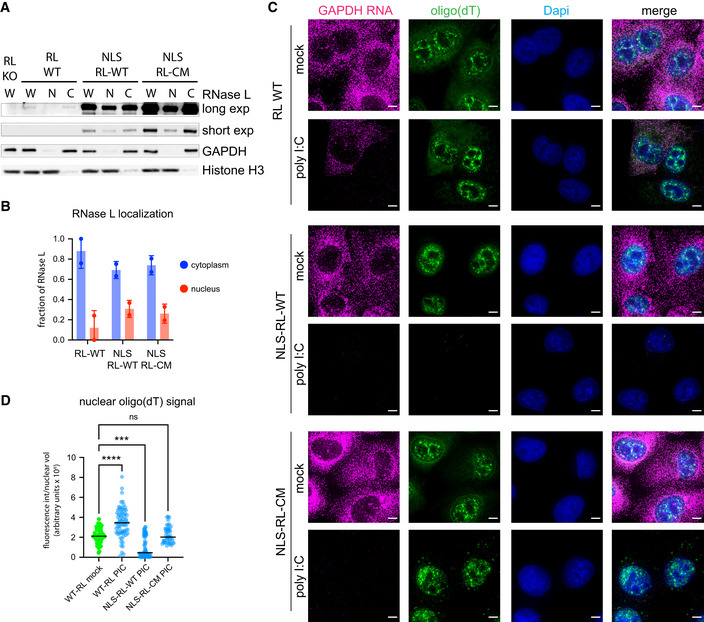
Nuclear‐localized RNase L degrades nuclear RNA in response to poly(I:C) Western analysis of nuclear (N) or cytoplasmic (C) fractions from whole cell lysates (W) from A549 cells (RL WT), RNase L knock out A549 cells (RL KO), or A549 cells with either wild‐type RNase L (NLS‐RL‐WT) or RNase L‐R667A (NLS‐RL‐CM) fused to a nuclear localization signal sequence. Short and long exposure with anti‐RL antibody. GAPDH protein used as cytoplasmic marker. Histone H3 protein used as nuclear marker.Graph depicting the fraction of RNase L found in the nucleus or cytoplasm. Mean and SEM of two experiments with individual experiment values plotted.FISH with probes to GAPDH mRNA or oligo(dT) to detect poly(A)+ RNA in A549 cells without or with either NLS‐RL‐WT or NLS‐RL‐CM either mock transfected or transfected with poly(I:C) for 4 h. Scale bar 5 micron.Graph depicting median and the value of the intensity of oligo(dT) fluorescence in individual nuclei divided by nuclear volume in the indicated cell types either mock transfected or transfected with poly(I:C) for 4 h. At least 43 nuclei analyzed per condition. Kruskal–Wallis test and Dunn’s multiple comparisons test. ****P‐value ≤ 0.0001, ***P‐value 0.0001, ns not significant. Source data are available online for this figure.

We fused the cmyc nuclear localization signal (NLS) to RNase L, introduced the modified wild‐type RNase L (NLS‐RL‐WT) and RNase L‐R667A catalytic mutant (NLS‐RL‐CM) into A549 cells via lentiviral transduction, and determined if the NLS was sufficient to target RNase L to the nucleus. By biochemical fractionation, we observed that approximately 30% of the NLS‐RL‐WT and NLS‐RL‐CM proteins were detected in the nuclear fraction, while 90% or more of the endogenous RNase L is in the cytosol (Fig 2A and B). This demonstrates that the NLS was sufficient to target a portion of RNase L to the nucleus. The exogenous RNase L proteins were overexpressed > 40 fold compared to the endogenous protein, which should allow it to robustly degrade both nuclear and cytoplasmic RNAs.

By examining RNAs by FISH, localization of RNase L to the nucleus resulted in RNA degradation in the nucleus. Specifically, nuclear poly(A) signal was depleted in cells expressing NLS‐RL‐WT (Fig 2C and D) in response to poly(I:C) treatment. The degradation of nuclear poly(A)+ RNAs was dependent on the catalytic activity of RNase L since cells expressing NLS‐RL‐CM did not degrade nuclear RNA (Fig 2C and D). In cells expressing only endogenous RNase L, nuclear poly(A) signal increased in response to poly(I:C) treatment (Fig 2C and D) which is consistent with the observation that activation of RNase L leads to inhibition of RNA export from the nucleus (Burke *et al*, 2021). Consistent with the tagged RNase L being in both the nucleus and cytosol, and the presence of the endogenous cytosolic RNase L, cytosolic RNA degradation as assessed by loss of the GAPDH mRNA occurred following treatment with poly(I:C) in cells expressing either form of the NLS‐RL fusion proteins (Fig 2C). Thus, this system allows for the degradation of nuclear RNA in living cells in an inducible fashion. We then used this approach to determine if nuclear RNP granules require RNA to maintain their structural integrity.

### Nucleolar structure is dependent on the presence of RNA

**Figure 3:**
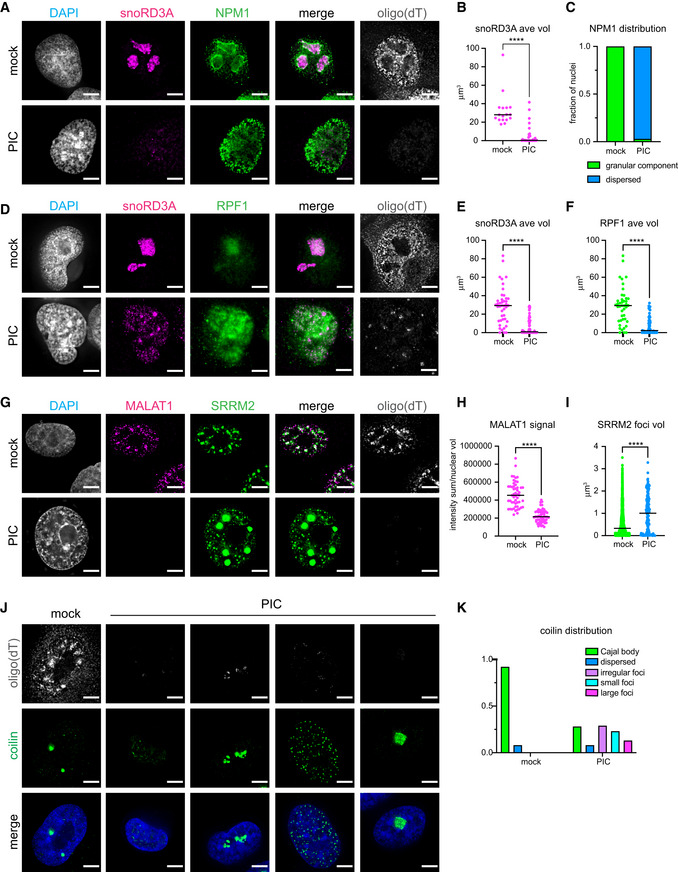
The structure of nucleoli, nuclear speckles and Cajal bodies is dependent on nuclear RNA Analysis of nuclear RNA granules in A549 cells with RNase L targeted to the nucleus (NLS‐RL‐WT) either mock transfected or treated with poly(I:C) (PIC) for 4 to 5 h. FISH analysis to detect poly(A)+ RNA and nucleolar‐localized snoRD3A RNA. IF analysis to detect nucleolar granular component protein nucleophosmin (NPM1).Graph of the mean and value of the average volume of individual snoRD3A foci in nuclei. 17 nuclei mock‐treated cells, 31 nuclei PIC‐treated cells.Fraction of nuclei with NPM1 protein enriched in ring structures classified as granular component assemblies or dispersed in nucleoplasm. 17 nuclei mock‐treated cells, 31 nuclei PIC‐treated cells.IF analysis to detect dense fibrillar component protein, ribosome processing factor 1 (RPF1).Graph of the mean and value of the average volume of individual snoRD3A foci in nuclei. 43 nuclei mock‐treated cells, 69 nuclei PIC‐treated cells.Graph of the mean and value of the average volume of RPF1 foci in 43 mock‐treated cells and 69 nuclei PIC‐treated cells.FISH analysis to detect poly(A)+ RNA and nuclear speckle‐localized MALAT1 RNA and IF analysis with anti‐sc35 antibody to detect nuclear speckle protein SRRM2.Graph of the mean and value of the intensity of MALAT1 signal divided by the nuclear volume of individual nuclei. 51 nuclei mock‐treated cells, 56 nuclei PIC‐treated cells.Graph of the median and value of the volume of individual SRRM2 foci in nuclei. Mock 1526 SRRM2 foci in 51 nuclei. PIC 136 SRRM2 foci in 20 nuclei that contain ≤ 10 SRRM2 foci.FISH analysis to detect poly(A)+ RNA and IF analysis to detect Cajal body protein coilin.Graph depicting the fraction of nuclei in mock‐ and PIC‐treated cells with decreased oligo(dT) with different distributions of coilin protein. 90 nuclei in mock‐treated cells, 192 nuclei in PIC‐treated cells. Data information: All images scale bar 5 microns. All graphs with significance testing. Mann–Whitney two‐tailed test. ****P‐value ≤ 0.0001. Source data are available online for this figure.

**Figure EV3:**
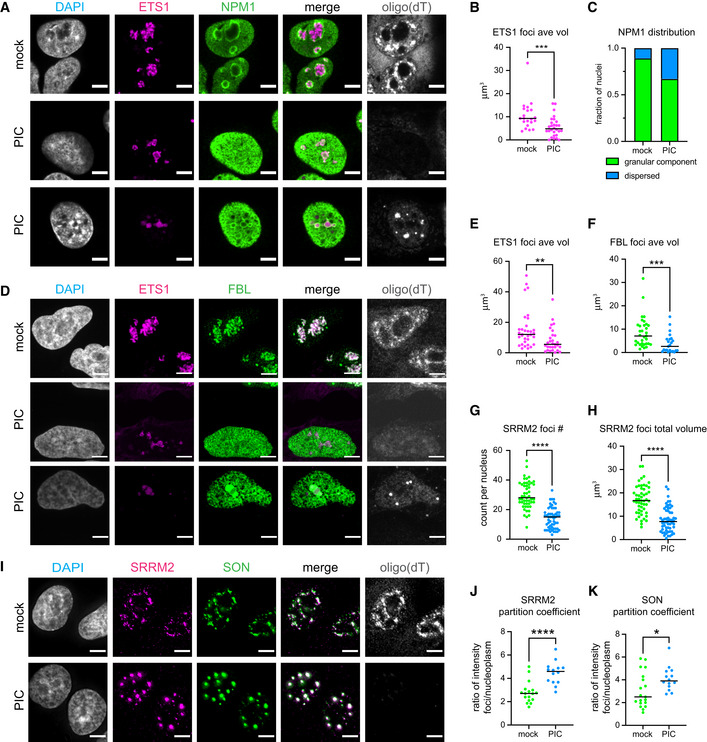
Effect of activation of RNase L in the nucleus on precursor rRNA and nucleolar proteins and on nuclear speckles Analysis of A549 cells expressing nuclear‐localized wild‐type RNase L mock or poly(I:C) treated for 5 h. ETS1 and oligo(dT) FISH and IF analysis of NPM1 protein.Graph of the mean and individual values for the average volume of ETS1 FISH signal in nuclei. ***P‐value = 0.0009. Mock 21 nuclei. PIC 28 nuclei.Fraction of nuclei with NPM1 protein enriched in ring structures of any size classified as granular component assemblies or dispersed in nucleoplasm. Mock 21 nuclei. PIC 24 nuclei in which ETS1 signal was lower than the median ave vol in mock‐treated cells.ETS1 and oligo(dT) FISH and IF analysis of FBL protein.Graph of the mean and individual values for the average volume of ETS1 FISH signal in nuclei. Mock 33 nuclei. PIC 29 nuclei. **P‐value = 0.0025.Graph of the mean and individual values for the average volume of fibrillarin (FBL) foci in nuclei. Mock 33 nuclei. PIC 23 nuclei in which ETS1 signal was lower than the median ave vol in mock‐treated cells. ***P‐value = 0.0008.Graph of the mean and individual value for the number of SRRM2 foci per nucleus. ****P‐value ≤ 0.0001 Mock 51 nuclei. PIC 56 nuclei.Graph of the mean and individual value for the total volume of SRRM2 foci divided by the nuclear volume of individual nuclei. ****P‐value ≤ 0.0001 Mock 51 nuclei. PIC 56 nuclei.Oligo(dT) FISH and IF analysis of two nuclear speckle proteins, SRRM2 and SON.Graph of the median and individual value of the ratio of SRRM2 fluorescence intensity in foci compared to in the nucleoplasm. ****P‐value ≤ 0.0001 Mock 17 foci in 5 nuclei. PIC 14 foci in 5 nuclei.Graph of the median and individual value of the ratio of SON fluorescence intensity in foci compared to in the nucleoplasm. *P‐value = 0.034 Mock 19 foci in 5 nuclei. PIC 14 foci in 5 nuclei. Data information: Scale bar 5 micron. All graphs with significance test Mann–Whitney two‐tailed test. Source data are available online for this figure.

The nucleolus is a large structure in the nucleus where transcription and processing of ribosomal RNAs and their assembly into ribosomal subunits occurs. It is organized into distinct subdomains, a fibrillar center (FC) containing rDNA, the dense fibrillar component (DFC) containing the precursor rRNA, and snoRNAs involved in initial steps in rRNA processing and the granular component (GC) where further processing, modification, and assembly of ribosomal subunits is thought to occur (Lam *et al*, 2005). To monitor if nucleolar‐associated RNA was accessible to degradation by RNase L, we used probes for the small nucleolar RNA, snoRD3A, a C/D box snoRNA which localizes to the DFC and GC (Gerbi & Brovjagin, 1997) and the 5’ end of the nascent 47S rRNA (ETS1) which is found in the DFC (Yao *et al*, 2019). In the mock‐transfected cells containing NLS‐RL‐WT, snoRD3A and ETS1 signal was in large nuclear structures (Figs 3A and D EV3A and D). snoRD3A signal was depleted in cells with activated RNase L in the nucleus (Fig 3A and B and, D and E), which we identified by depletion of nuclear oligo(dT) signal (Fig 3A). The ETS1 signal was also reduced by nuclear RNase L activation (Fig EV3A and B, and D, and E); however, it was not as strongly depleted as snoRD3A perhaps due to its structure, protection by associated proteins, or continual production by transcription.

Examination of NPM1 (nucleophosmin 1), which is enriched in the GC of nucleoli (Spector *et al*, 1984), demonstrated that the GC component of nucleoli is lost when nuclear RNA is degraded. In the mock‐transfected cells, NPM1 was found in the nucleoplasm and concentrated in a ring surrounding the snoRD3A and ETS1 RNA signal (Figs 3A and EV3A). In cells in which nuclear RNA was degraded, based on depletion of oligo(dT) and snoRD3A RNA signal, NPM1 was dispersed in the nucleoplasm (Fig 3A and C), suggesting that the granular component of the nucleolus had been disrupted. Similar disruption of the granular component was observed in cells with reduced ETS1 foci although NPM1 was also seen to form rings around small residual ETS1 structures (Fig EV3A and C).

**Figure EV4:**
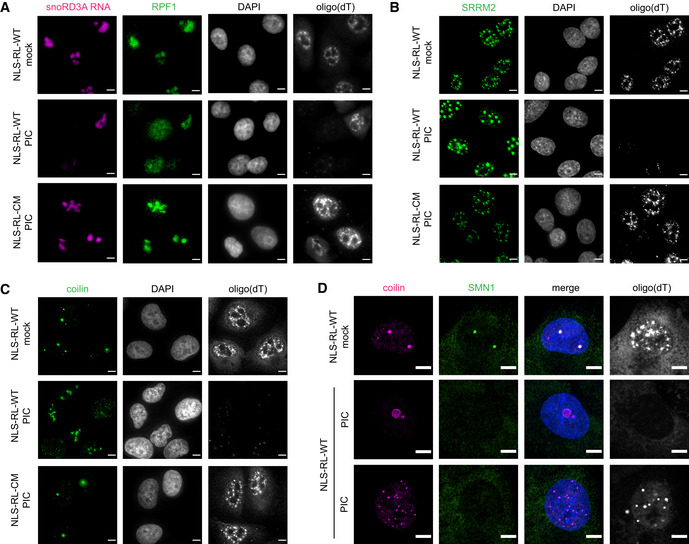
Changes in nuclear RNA granule morphology is dependent on loss of nuclear RNA and SMN1 disperses into nucleoplasm in response to nuclear RNA degradation IF analysis of nuclear RNA granule proteins in A549 cells expressing nuclear‐localized wild‐type RNase L or catalytic mutant RNase L‐R667A mock transfected or treated with poly(I:C) for 5 h. FISH analysis to detect nucleolar‐localized snoRD3A RNA and poly(A)+ RNA. IF analysis of nucleolar protein RPF1.Oligo(dT) FISH and IF analysis of nuclear speckle protein SRRM2 with sc35 antibody.Oligo(dT) FISH and IF analysis of Cajal body protein coilin.Oligo(dT) FISH and IF analysis of Cajal body protein coilin and SMN1. Data information: Scale bar 5 microns.

We also examined how the dense fibrillar component of nucleoli was affected by nuclear RNA degradation by analyzing the localization of two DFC proteins, RPF1 (ribosome production factor 1) and FBL (fibrillarin). RPF1 binds to pre‐60S ribosomal subunits (Wehner & Baserga, 2002; Kater *et al*, 2017). FBL is a component of snoRNPs involved in the first steps of processing pre‐rRNA and was recently found to be important for sorting the nascent 47S pre‐rRNA into the DFC (Yao *et al*, 2019). RPF1 and FBL colocalized with the snoRD3A RNA or the ETS1 signal in the mock‐transfected cells (Figs 3D and EV3D). In the majority of cells with reduced snoRD3A levels, RPF1 was dispersed in the nucleoplasm (Fig 3D and F) consistent with the dense fibrillar component of nucleoli requiring RNA for its structure. We did observe that in 30% of the cells in which the snoRD3A RNA was depleted, RPF1 was concentrated in one or a few large foci as well as being found in the nucleoplasm. These residual RPF1 assemblies could be arising in nuclei where RNA degradation of the pre‐rRNA is not as robust. No change in RPF1 localization was observed in response to poly(I:C) in cells expressing the nuclear‐targeted catalytic mutant form of RNase L (Fig EV4A) consistent with the changes in nucleolar structure being dependent on the degradation of nucleolar RNA.

Changes in the DFC were also observed when we examined fibrillarin in response to nuclear RNA degradation. The average size of FBL foci was reduced in nuclei with reduced ETS1 signal with some nuclei having FBL dispersed in the nucleoplasm (Fig EV3D–F). The residual FBL foci and ETS1 foci did not have the fibrillar appearance seen in mock‐treated cells being more rounded instead (Fig EV3D). Taken together, these findings suggest that RNA is required to maintain the overall structure of nucleoli. The requirement for RNA for nucleolar structure is consistent with previous work showing transcription of rRNA is required for nucleolar formation (Benavente, 1991).

### The size and shape of nuclear speckles changes following nuclear RNA degradation

Mammalian cells contain 10–50 irregular‐shaped nuclear bodies referred to as nuclear speckles that are found in interchromatin spaces within the nucleus (Spector & Lamond, 2011). Nuclear speckles contain poly(A)+ RNA and factors involved in mRNA metabolism, including snRNPs, splicing factors, RNA export and quality control factors, subunits of RNA polymerase II, transcription factors, and translation initiation factors (Hall *et al*, 2006; Spector & Lamond, 2011). We monitored MALAT1 RNA, a lncRNA found in nuclear speckles (Hutchinson *et al*, 2007), to determine if RNA in speckles is susceptible to degradation by RNase L. The SC35 antibody, which recognizes the SRRM2 protein (Ilik *et al*, 2020), was used to monitor the effect of nuclear RNA depletion on nuclear speckles.

We observed that degradation of nuclear RNA, including MALAT1, led to a dramatic rearrangement of nuclear speckles. Specifically, in mock‐treated cells, we observed polyA+ RNA and SRRM2 protein in discrete nuclear speckles that were often associated with local concentrations of MALAT1 (Fig 3G). In response to poly(I:C) treatment, MALAT1 and nuclear polyA+ RNA was depleted in cells expressing nuclear‐localized RNase L (Fig 3G and H). Strikingly, the degradation of nuclear RNA corresponded with the loss of nuclear speckles and re‐organization of SRRM2 into a few large discrete nuclear assemblies (Figs 3G–I and EV3G–I). Similar changes in the localization of a second nuclear speckle protein, SON, were observed in response to nuclear RNA degradation (Fig EV3I). No change in SRRM2 localization was observed in response to poly(I:C) in cells expressing the nuclear‐targeted catalytic mutant RNase L (Fig EV4B). Since MALAT1 is not required for nuclear speckles, and depletion of MALAT1 from speckles does not result in large round bodies (Tripathi *et al*, 2010; Fei *et al*, 2017), we infer that RNAs other than MALAT1 determine the morphology of nuclear speckles. The change in size and morphology of nuclear speckles in response to nuclear RNA degradation suggests that RNA normally limits the assembly of SRRM2 and SON, and potentially other speckle components, into larger structures.

### Coilin, a Cajal body protein, re‐localizes in response to nuclear RNA degradation

Cajal bodies are sub‐compartments in the nucleus involved in the assembly and RNA modification of snRNP complexes required for pre‐mRNA splicing, ribosome biogenesis, histone mRNA processing, and telomere synthesis (Staněk, 2017; Machyna *et al*, 2013). In addition to these substrate snRNP complexes, Cajal bodies contain many protein factors as well as small Cajal body‐specific RNAs (scaRNAs) (Machyna *et al*, 2013). Cajal bodies form at endogenous clusters of snRNA genes (Frey & Matera, 1995; Smith *et al*, 1995; Gao *et al*, 1997) and can be nucleated in a transcription‐dependent manner at artificial arrays containing snRNA or scaRNA genes (Frey *et al*, 1999; Kaiser *et al*, 2008). To determine if RNA is also important for maintaining Cajal body assembly, we examined the organization of Cajal bodies in response to the degradation of nuclear RNA.

We observed that nuclear RNA degradation led to dramatic rearrangement of the Cajal body protein coilin. We have been unable to detect Cajal body‐associated RNAs reliably by FISH; therefore, we used the loss of oligo(dT) signals as a means to identify cells in which nuclear RNA was degraded. However, since RNase L is a broad non‐specific nuclease that cleaves tRNAs, rRNAs, and mRNAs in the cytosol (Donovan *et al*, 2017, Burke *et al*, 2019; Rath *et al*, 2019) and snoRNAs (Fig 2A and B, and D, and E), lincRNAs (Fig 2G and H), and pre‐rRNAs (Fig EV3A and B, and D, and E) in the nucleus, we infer that Cajal body‐associated RNAs are also degraded. In mock‐treated cells, coilin was localized to a few foci per nucleus (Fig 3J). In contrast, in cells in which nuclear RNA was degraded, coilin was found in a variety of different types of assemblies (Fig 3J and K). Similar to mock‐treated cells, in 8% of the nuclei with reduced oligo(dT) signal, coilin was dispersed in the nucleoplasm (Fig 3J, first PIC panel). Coilin was in irregular‐shaped foci of varying sizes and number (Fig 3J, second PIC panel) in 29% of the nuclei. In 23% of the nuclei in which RNA had been degraded, coilin was localized to many small foci that may represent sub‐assemblies of Cajal bodies (Fig 3J, third PIC panel). In 13% of nuclei, coilin formed one or two large irregular‐shaped foci (Fig 3J, fourth PIC panel). In the remaining nuclei in which poly(A)+ RNA had been degraded, coilin was found in foci that resembled Cajal bodies in size and number. These foci could be Cajal bodies in which the resident RNAs have not been fully degraded. The distribution of coilin did not change in response to poly(I:C) treatment in the cells expressing NLS‐RL‐CM (Fig EV4C). Assuming coilin represents the behavior of Cajal bodies, we interpret these results to argue that nuclear RNA, presumably one or more of the RNA species that are associated with Cajal bodies, is required to maintain their integrity.

Consistent with Cajal bodies requiring RNA for their integrity, examination of the SMN1 protein, which can bind directly to coilin (Hebert *et al*, 2001) and localizes with Cajal bodies in addition to gems (Liu & Dreyfuss, 1996), showed that upon nuclear RNA degradation SMN1 re‐localized from Cajal bodies to diffuse nuclear distribution (Fig EV4D). This observation argues that the interaction of SMN1 with coilin is disrupted when RNA is depleted.

RNase L activation did not lead to apparent changes in either the nuclear lamin network or promyelocytic leukemia (PML) bodies which are nuclear condensates that do not contain RNA (Boisvert *et al*, 2000) (Fig EV2D–G). This observation is consistent with degradation of RNA in the nucleus specifically affecting RNA‐dependent assemblies.

### Protein components of nuclear RNA granules do not co‐assemble when nuclear RNA is degraded

We observed that the assemblies of the protein components of Cajal bodies, nuclear speckles, and the nucleolus that formed when nuclear RNA was degraded were often located in regions of the nucleus that were depleted in chromatin (Figs 3 and EV3 and EV4). This observation suggested that the proteins of these different RNA granules could assemble or possibly aggregate together in the absence of their resident RNAs. We performed immunofluorescence analysis to determine if the protein components of the different nuclear RNA granules colocalized when nuclear RNA degradation was induced.

**Figure 4:**
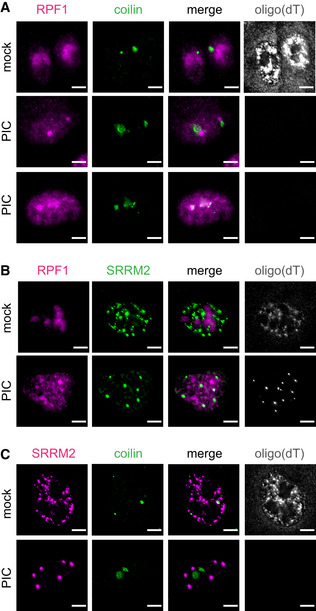
Protein components of nuclear RNA granules do not co‐assemble when nuclear RNA is degraded Analysis of nuclear RNA granules in A549 cells with RNase L targeted to the nucleus (NLS‐RL‐WT) either mock transfected or treated with poly(I:C) (PIC) for 5 h. Oligo(dT) FISH and IF analysis of nucleolar protein RPF1 and Cajal body protein coilin.Oligo(dT) FISH and IF analysis of nucleolar protein RPF1 and nuclear speckle protein SRRM2.Oligo(dT) FISH and IF analysis of Cajal body protein coilin and nuclear speckle protein SRRM2. Data information: Scale bar 5 microns.

In cells in which nuclear RNA had been degraded and that contained both coilin and RPF foci, coilin tended to be in close proximity with RPF1 foci (Fig 4A middle panel). These coilin assemblies may be similar to structures referred to as nucleolar caps that contain coilin and form next to nucleoli when transcription is inhibited (Shav‐Tal *et al*, 2005). The distinct nature of the coilin and RPF1 foci is consistent with these proteins, either individually or in combinations with other proteins, maintaining sufficient information to allow the formation of specific condensates. However, in some cells, coilin overlapped with RPF1 signal (Fig 4A bottom panel). Coilin has been previously found in nucleoli under varying cellular perturbations (Trinkle‐Mulcahy & Sleeman, 2017) suggesting that it has some affinity to nucleolar components and may contain a putative nucleolar localization sequence.

The assemblies containing the nuclear speckle protein SRRM2 did not co‐localize with the foci containing the Cajal body protein coilin or the nucleolar protein RPF1 (Fig 4B and C). Thus, the alternative nuclear speckle condensates that form in response to nuclear RNA degradation represent another distinct assembly.

The ability of some protein components of RNP granules to form novel assemblies in response to RNA degradation is consistent with these proteins having self‐assembly properties either individually or with other proteins. In the cases where we have observed *de novo* assemblies after RNA degradation, the proteins forming self‐assemblies are thought to play roles in the assembly of their RNP granules (Hebert & Matera, 2000; Ilik *et al*, 2020). The observation that these new RNase‐induced assemblies are more spherical is consistent with the hypothesis that RNA can limit the self‐assembly of some RNA‐binding proteins.

If RNA limits the self‐assembly of some RNA‐binding proteins, then the protein‐only assemblies that form upon RNA degradation would be predicted to have an increased partition coefficient for their protein components. To test this possibility, we measured the partition coefficients of SRRM2 and SON into nuclear speckles and into their novel assemblies that form upon RNA degradation. We observed that both SRRM2 and SON proteins showed increased partitioning into assemblies following RNA degradation (Fig EV3J and K). This argues RNA limits the self‐assembly of SRRM2 and SON and provides evidence for RNA limiting the formation of some protein‐based assemblies.

### Reduction of nuclear RNA does not lead to FUS self‐assembly

In principle, RNA could be limiting the assembly of RNA‐binding proteins to prevent the formation of inappropriate assemblies. This idea was suggested by the observation that FUS, an RNA‐binding protein with IDRs, formed assemblies after RNase A was injected into nuclei (Maharana *et al*, 2018). To examine if FUS behaved in a similar way after RNase L degradation of nuclear RNA, we monitored the distribution of FUS in cells with the NLS‐RL‐WT protein after poly(I:C) treatment.

**Figure 5:**
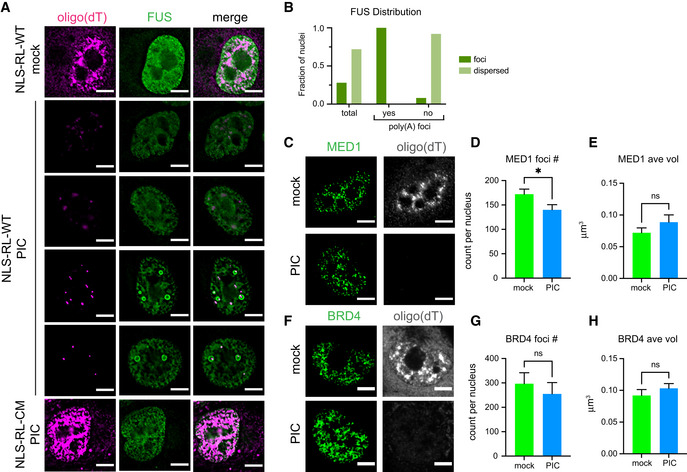
Analysis of the effect of nuclear RNA degradation on super‐enhancer condensates and FUS assembly Analysis of A549 cells with NLS‐RL‐WT or NLS‐RL‐CM either mock transfected or treated with poly(I:C) (PIC) for 5 h. FISH analysis to detect poly(A)+ RNA and IF analysis against FUS protein. Images are a single 0.2 micron Z section.Graph depicting the fraction of nuclei with FUS dispersed in the nucleoplasm or in foci in cells with reduced nuclear oligo(dT) signal. All the nuclei (total) in which nuclear RNA was degraded (*N* = 64) or the same nuclei classified as to whether or not they contained residual poly(A) foci. Nuclei with poly(A) foci (*N* = 14). Nuclei without poly(A) foci (*N* = 50).FISH analysis to detect poly(A)+ RNA and IF analysis against mediator complex protein, MED1, to detect super‐enhancer condensates.Number of MED1 foci in mock‐ and PIC‐treated NLS‐RL‐WT cells.Average volume of individual MED1 foci in mock‐ and PIC‐treated NLS‐RL‐WT cells.FISH analysis to detect poly(A)+ RNA and IF analysis against BRD4, to detect super‐enhancer condensates.Number of BRD4 foci in mock‐ and PIC‐treated NLS‐RL‐WT cells.Average volume of individual BRD4 foci in mock‐ and PIC‐treated NLS‐RL‐WT cells. Data information: Scale bar 5 microns. (D, E) Mean and SEM of three independent experiments with at least 10 cells of each cell type and condition per experiment. (G, H) Mean and SEM of three independent experiments with 8‐13 cells of each cell type and condition per experiment. (D, E, G, and H) Unpaired two‐tailed *t*‐test, *P‐value = 0.02, ns, non‐significant. Source data are available online for this figure.

We observed that FUS distribution was largely unchanged in the majority of cells with efficient nuclear RNA degradation. Specifically, FUS was dispersed throughout the nucleoplasm in the mock‐treated A549 cells (Fig 5A), and in 70% of cells responding to poly(I:C) treatment, we observed robust nuclear RNA degradation and FUS remained dispersed in the nucleoplasm (Fig 5A and B). This observation indicates that reduction of nuclear RNA does not lead to FUS self‐assembly, at least at endogenous expression levels.

Interestingly, in 30% of the cells with reduced nuclear oligo(dT) signal, residual poly(A) foci appeared to nucleate FUS assemblies since FUS assemblies either overlapped with the poly(A) foci or assembled on their surface (Fig 5A and B). The assembly of the FUS foci was dependent on nuclear RNA degradation given that they were not observed after poly(I:C) treatment in cells with the catalytic mutant form of RNase L in the nucleus (Fig 5A). The observation that the FUS assemblies that form in the nucleus when RNA is degraded contain some type of RNA is consistent with these FUS assemblies requiring at least a low level of RNA. This suggests a model where FUS and RNA can assemble a condensate under certain concentrations of RNA and protein, with RNA being a required component of that assembly.

### Nuclear RNA degradation does not dramatically alter super‐enhancer condensates

Recently, transcription factors and coactivators of transcription have been found to form condensates in the nucleus at the sites of super‐enhancers (Boija *et al*, 2018; Sabari *et al*, 2018). Super‐enhancers are clusters of enhancers that drive the robust expression of genes by assembling a high density of transcriptional machinery. Transcription of super‐enhancers produces high quantities of what are referred to as enhancer RNAs or eRNAs (Hah *et al*, 2015). eRNAs have been proposed to promote enhancer function by playing a role in the phase separation of transcriptional components (Arnold *et al*, 2020), while high levels of RNA from nascent transcription are proposed to inhibit the formation of super‐enhancer condensates (Henninger *et al*, 2021). We therefore examined whether super‐enhancer condensates are altered when nuclear RNA is degraded by monitoring MED1, a component of the coactivator Mediator complex, and the transcriptional coactivator BRD4 which have both been used as markers for transcriptional condensates (Sabari *et al*, 2018).

We observed that transcriptional condensates showed little alterations when nuclear RNA was degraded. MED1 and BRD4 were found in many small foci in the nucleus in mock‐treated cells (Fig 5C–G). There was a tendency for nuclear RNA degradation to lead to a small decrease in the number of MED1 and BRD4 foci, although the effect was only significant for MED1 foci (Fig 5D and G). There was also no significant change in the average volume of MED1 and BRD4 foci in NLS‐RL‐WT cells in which nuclear RNA had been degraded (Fig 5E and H).

**Figure EV5:**
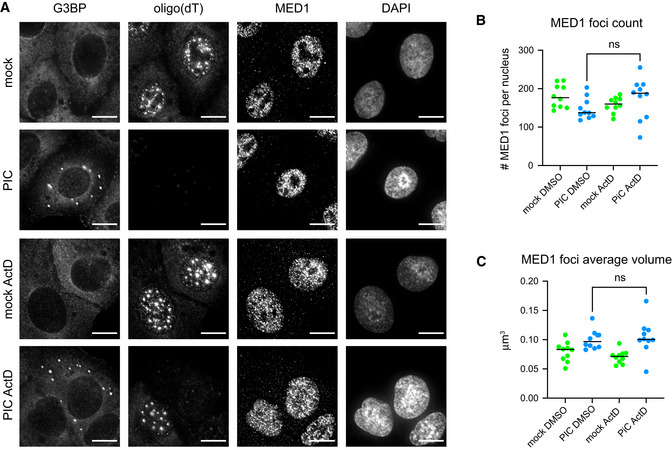
Inhibition of transcription in combination with nuclear RNA degradation did not affect the number or volume of MED1 foci IF analysis of super‐enhancer condensate protein MED1 in A549 cells expressing nuclear‐localized wild‐type RNase L mock transfected or treated with poly(I:C) with DMSO or 1 μg/ml Actinomycin D (ActD) for 5 h. IF against G3BP used to monitor cells responding to poly(I:C), FISH analysis for poly(A)+ RNA used to monitor nuclear RNA degradation and transcription inhibition. Scale bar 5 microns.Number of MED1 foci per cell in mock‐, PIC‐, ActD‐, or PIC and ActD‐treated cells.Average volume of individual MED1 foci per cell in mock‐, PIC‐, ActD‐, or PIC and ActD‐treated cells. Data information: (B, C) Graphs depict the mean and individual values for each cell. At least 10 cells in each condition were counted. Unpaired, two‐tailed *t*‐test, ns non‐significant. Source data are available online for this figure.

The lack of a large effect of nuclear RNA degradation on transcriptional condensates could be due to continued transcription of short‐lived eRNAs or mRNAs. To test this possibility, we treated cells with Actinomycin D to inhibit transcription while we induced nuclear RNA degradation. Transcription inhibition in combination with nuclear RNA degradation did not significantly affect the number of MED1 foci or alter their average size or morphology (Fig EV5A and B). The observation that nuclear RNA degradation coupled with transcriptional inhibition did not disrupt MED1 foci suggests that eRNAs are not required to maintain transcription condensates.

## Discussion

A striking conclusion from this work is that the majority of RNP granules examined require RNA for their proper morphology and structure. The key observation is that degradation of either cytosolic or nuclear RNAs by activation of RNase L leads to loss of resident RNAs in these organelles and alterations in their morphology as judged by the localization of protein components. Since RNA affects the assembly of SGs, Cajal bodies, nuclear speckles, and the nucleolus, we suggest that RNA molecules play a broad role in determining membrane‐less compartments in eukaryotic cells.

The requirement for RNA in the formation of nuclear RNP granules is consistent with earlier work showing that nuclear RNP granules generally form near the sites of transcription of their resident RNAs (Arias Escayola & Neugebauer, 2018). For example, the nucleolus forms around transcribing pre‐ribosomal RNA (rRNA) molecules (Falahati *et al*, 2016). Cajal bodies are found near snRNA genes (Frey & Matera, 1995; Smith *et al*, 1995; Gao *et al*, 1997), paraspeckles form on the NEAT1 gene (Mao *et al*, 2011b), and the histone locus body (HLB) is found assembled on the arrays of histone genes (Liu *et al*, 2006). In each of these cases, the arrayed nature of these genes creates a very high local concentration of the specific RNA leading to a unique RNP granule formation. The formation of the nuclear RNP granule can be understood as being initially defined by the high concentration of specific transcripts (e.g., pre‐rRNA, or snRNAs) that then recruit additional RNA‐binding proteins and interacting RNAs (such as snoRNAs to the nucleolus) to complete the RNP granule formation. Nuclear speckles have a more heterogeneous composition but appear to form from the same principles. Nuclear speckles are thought to be surrounded by active genes that deliver nascent transcripts directly into nuclear speckles (Quinodoz *et al*, 2018, 2021), and this high concentration of nascent transcripts can recruit RNA‐binding proteins and snRNAs for RNA processing. In the extreme view, every transcription unit can be thought of as generating a transient RNP granule through the production of multiple copies of the same transcript with large easily identified RNP granules forming on arrayed genes, or loci with RNAs that are retained in the vicinity of the gene by yet to be defined mechanisms. In this regard, it is notable that many lncRNAs appear to define unique nuclear RNP granules that can modulate chromatin structure (Quinodoz *et al*, 2021).

The requirement for RNA for the integrity of SGs *in vivo* is consistent with *in vitro* studies. Although the purification protocol for SGs enriches for substructures which are stable when treated with RNase (Jain *et al*, 2016), this is due to the RNA in these substructures being inaccessible to RNases once cells are lysed (Van Treek *et al*, 2018). The importance of RNA to SG assembly is supported by recent studies in which SG‐like structures are assembled from cell lysates/cell fractions in which the formation of these structures is induced by the addition of RNA (Begovich & Wilhelm, 2020) and inhibited by RNase treatment of lysates before assemblies are induced (Freibaum *et al*, 2021). In contrast to what we observed *in vivo*, PBs extracted from yeast cells disassemble when treated with RNase (Teixeira *et al*, 2005). This suggests that the concentration of PB protein components in the cytoplasm is sufficient to maintain an interaction network capable of preserving the integrity of PBs.

**Figure 6:**
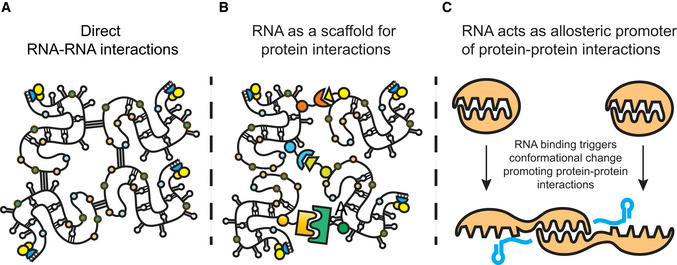
Model for the potential roles of RNA in the assembly and maintenance of membrane‐less organelles containing RNA and protein Direct RNA‐RNA interactions.RNA as a scaffold for protein interactions.RNA acts as allosteric promoter of protein‐protein interactions.

In principle, RNA molecules can promote membrane‐less organelle assembly in three manners (Fig 6). First, RNA molecules could allow for intermolecular RNA–RNA interactions as suggested for the assembly of SGs (Fig 6A) (Van Treek *et al*, 2018; Tauber *et al*, 2020). Alternatively, RNA molecules could provide scaffolding to increase the number and valency of proteins able to engage in intermolecular interactions (Fig 6B) as the case for the function of NEAT 1 RNA in the assembly of paraspeckles (Clemson *et al*, 2009; Mao *et al*, 2011; West *et al*, 2016). Finally, RNA molecules could serve as allosteric co‐factors to alter the assembly properties of proteins that could drive organelle assembly (Fig 6C). We anticipate that evolution will have made use of all these mechanisms in different contexts.

For the RNA granules that are sensitive to the loss of RNA, we observe two different outcomes of their protein components with different implications. First, the proteins can distribute widely in their cellular compartment. For example, NPM1, and in many nuclei, RPF1 become widely distributed in the nucleoplasm when nucleolar RNAs are degraded (Fig 4). This dispersion of the marker protein argues that specific protein is insufficient to form any type of membrane‐less assembly in the absence of RNA, and therefore can be inferred to have limited protein‐based assembly parameters.

In other cases, we observed degradation of resident RNAs in a membrane‐less organelle led to the formation of a new type of assembly. For example, we have previously shown that when preformed SGs disassemble in response to cytoplasmic RNA degradation, a residual membrane‐less organelle, an RLB, remains with a distinctly different protein composition (Burke *et al*, 2020). Alternatively, in cells with complete nuclear RNA degradation, as assessed by oligo(dT) staining in the nucleus, coilin forms either a few large or multiple small irregular‐shaped assemblies (Fig 4). Similarly, when nuclear RNA is degraded, nuclear speckle proteins, SRRM2 and SON, form several large spherical assemblies (Fig 4). Although we cannot rule out the possibility that these new assemblies are being nucleated by residual RNA of some kind, it is likely that they reveal self‐assembly properties of these proteins by themselves, or in conjunction with other nuclear proteins. Consistent with these proteins having self‐assembly properties, coilin is known to self‐assemble (Hebert & Matera, 2000), and SON and SRRM2 have extended IDRs and play a combinatorial role in the formation of nuclear speckles (Ilik *et al*, 2020).

Interactions with resident RNA molecules within granules or with RNA outside of their respective granules could limit proteins from associating with each other, and in the absence of RNA, such protein‐based assemblies can then accumulate. Evidence in support of RNA limiting protein‐based assemblies in some cases is that the partition coefficients into the RNA‐independent assemblies of SRRM2 and SON increase when nuclear RNA is degraded (Fig EV3J and K). Consistent with RNA being required for the morphology of nuclear speckles, nuclear speckle components rearrange into similar large spheroid bodies when transcription is inhibited with ActD (Shopland *et al*, 2002; Shav‐Tal *et al*, 2005; Sasaki *et al*, 2009). This demonstrates a second general rule of RNA molecules in modulating the organization of the cell which is to limit assembly of protein‐driven large assemblies that might be unproductive for normal biological function.

We observed very little if any change in the number, size, or morphology of transcriptional condensates in response to nuclear RNA degradation. Super‐enhancers crowd transcription factors, mediator, and other transcriptional coactivators at densities that allow for condensates to form (Shrinivas *et al*, 2019). Enhancer RNAs and the nascent transcripts within transcriptional condensates are not thought to play structural roles in their assembly although they have been proposed to play a regulatory role in their formation and dissolution (Henninger *et al*, 2021). Our analysis did not test whether the dynamics of transcriptional condensates is affected by the depletion of RNA in the nucleus.

The differential sensitivity of assemblies to RNA degradation can be understood by the combination of molecular interactions promoting their formation. For example, transcriptional condensates are nucleated by the binding of transcriptional regulators to DNA, creating a high local concentration of proteins that can then recruit other components through protein–protein interactions. Given this set of molecular interactions, RNA would not be anticipated to be required for their formation and/or persistence. In contrast, RNP granules can be understood to assemble through a combination of protein–RNA, protein–protein, and RNA–RNA interactions (Van Treek and Parker, 2018). Only in cases where RNP granules are dominated by protein–protein interactions will the RNP granule be insensitive to RNA degradation. The high density of protein–protein interactions that contribute to PB formation (Jonas & Izaurralde, 2013) may explain why these structures can persist following RNA degradation. A molecular understanding of the manners by which RNA contributes to different RNP granules should further illuminate this issue.

## Materials and Methods

### Antibodies

For IF analysis: rabbit anti‐Dcp1(D2P9W) antibody (CellSignaling 13233S) 1:500, mouse anti‐DYKDDDDK tag(9A3) (Flag) antibody (Cell Signaling 8146S) 1:1,000, mouse anti‐G3BP antibody (Abcam ab56574) 1:1,000, mouse anti‐nucleophosmin (NPM1) (Abcam ab10530) 1:1,000, rabbit anti‐RPF1 (Sigma HPA024642) 1:100, mouse anti‐sc35 antibody (NovusBio NB100‐1774) 1:1,000, mouse anti‐coilin antibody (Abcam ab87913) 1:1,000, rabbit anti‐coilin antibody (Santa Cruz sc32860) 1:200, rabbit anti‐SRRM2 (Thermo Fisher/Invitrogen PA5‐66827) 1:200, rabbit anti‐SON (Thermo Fisher/Invitrogen PA5‐65108) 1:200, rabbit anti‐MED1 (Abcam ab64965) 1:500, mouse anti‐FUS antibody (atlas antibodies AMAb90549) 1:200, rabbit anti‐BRD4 (Abcam ab128874) 1:500, rabbit anti‐alpha tubulin (Abcam ab18251) 1:1,000, mouse anti‐pan keratin (Cell Signaling 4545T) 1:500, mouse anti‐lamin A/C (Cell Signaling 4777) 1:500, mouse anti‐PML (Abcam ab96051) 1:500, rabbit anti‐fibrillarin (Abcam ab5821) 1:500, mouse anti‐SMN1 (Cell Signaling 12976S) 1:400, goat anti‐rabbit IgG AF647 (Abcam ab150079) 1:1,000, and goat anti‐mouse IgG FITC (Abcam ab6785) 1:1,000.

For Western analysis: mouse anti‐RNase L antibody (Novus Biologicals NB 100‐351) 1:2,000, mouse anti‐GAPDH‐HRP (Santa Cruz Biotechnology sc47724) 1:5,000, rabbit anti‐Histone H3 antibody (Novus Biologicals NB 500‐171) 1:1,000, anti‐mouse IgG HRP (Sigma 4416) 1:5,000, anti‐rabbit IgG HRP (Cell Signaling 7074S) 1:5,000.

### Cell culture

A549 and A549 RL‐KO cell lines were described in Burke *et al* (2019). Cells were maintained at 5% CO_2_ and 37°C in Dulbecco’s modified eagle medium (DMEM) supplemented with fetal bovine serum (10% v/v) and penicillin/streptomycin (1% v/v). Cells were tested for mycoplasma contamination by the cell culture core facility during the study and were negative.

### Oligonucleotides

cmyc_NLS_RL_sen primer 5′‐CACCGGGACTGAAACTCGAGGGTACCGCCACCATGCCTGCTGCTAAGAGAGTGAAACTGGATGAGAGCAGGGATCATAACAA‐3′

cmyc_NLS_RL anti primer 5′‐ACAAGAAAGCTGGGTCTAGATTAGCACCCAGGGCTGGCCAACC‐3′

oCDRP498 5′‐CCATGACGGTGATTATAAAGATCATGACATCGACTACAAGGATGACGATGACAAGATGGAGGCGCTGAGTCG‐3′

oCDRP499 5′‐TAGGTTGTGGTTGTCTTTGTTCTTG‐3′

oCDRP500 5′‐ACAAAGACAACCACAACCTAATGGAGAGCAGGGATCATAACAAC‐3′

oCDRP501 5′‐ACAAGAAAGCTGGGTCTAGATCAGCACCCAGGGCTGGC‐3′

### Plasmids

The cmyc NLS was fused to the N‐terminus of RNase L and RNaseL‐R667A by PCR amplification using primers cmyc_NLS_RL_sen and cmyc_NLS_RL anti and the RNase L and RNaseL‐R667A pLenti‐EF1‐Blast plasmids described in Burke *et al* (2019) as templates. The cmyc‐NLS‐RNaseL and RNaseL‐R667A amplicons were inserted into Xho1/Xba1 cleaved pLenti‐EF1‐Blast vector using In‐Fusion (Clontech). pVSV‐G, pRSVRev, and pMDlg‐pRRE plasmids used for generation of lentiviral particles were described in Burke *et al* (2019). Flag‐tagged Dcp1a was fused to the RNase L by amplifying DCP1a using primers oCDRP498 and 499 and pcDNA3‐FLAG‐hDcp1a (Lykke‐Andersen, 2002) as a template and amplifying RNaseL and RNaseL‐R667A using primers oCDRP500 and 501 and the RNase L and RNaseL‐R667A pLenti‐EF1‐Blast plasmids described in Burke *et al* (2019) as templates. The DCP1 and RNaseL amplicons were inserted into Xho1/Xba1 cleaved pLenti‐EF1‐Blast vector using In‐Fusion (Clontech).

All insert sequences were confirmed by sequencing.

### Generation of lentiviral particles and stable cell lines

To generate the NLS‐RNase L, NLS‐RNase L‐R667A, DCP1‐RNase L, and DCP1‐RNase L‐R667A lentiviral particles, HEK293T cells (T25 flasks) were co‐transfected with 2.7 μg pLenti‐EF1‐cmycNLS‐RNase L‐Blast, pLenti‐EF1‐cmycNLS‐RNase L‐R667A‐Blast, pLenti‐EF1‐DCP1‐RNase L‐Blast or pLenti‐EF1‐ DCP1‐RNase L‐R667A‐Blast, 0.8 μg pVSV‐G, 0.8 μg pRSVRev, and 1.4 μg pMDLg‐pRRE using lipofectamine 2000 (Thermo Fisher). Medium was replaced 6 h post‐transfection. Medium was collected at 48 h post‐transfection and filter‐sterilized with a 0.45‐um filter. To create NLS‐RL‐WT and NLS‐RL‐CM A549 stable cell lines, A549 cells were seeded in T‐25 flasks, when 80% confluent, cells were incubated with 1 ml of NLS‐RNase L or NLS‐RNase L‐R667A lentiviral particles containing 10 μg of polybrene for 1 h with periodic rocking. Normal medium was then added to the flask and incubated for 24 h. Medium was removed 24 h post‐transduction and replaced with selective growth medium containing 5 μg/ml of Blasticidine S hydrochloride (Sigma‐Aldrich). Selective medium was changed every few days, then replaced with normal growth medium after 7 days. DCP1‐RNase L and DCP1‐RNase L‐R667A A549 RL‐KO stable cells lines were made in a similar manner.

### Cell fractionation and western blotting

Fractionation of A549, A549‐RL‐KO, A549 NLS‐RL‐WT, and A549 NLS‐RL‐CM cells was performed as described in Burke & Sullivan, 2017. Cells grown in one well of six‐well plate were trypsinized, equivalent number of cells were washed with phosphate‐buffered solution (PBS), resuspended in 200 μl of CSKT buffer [10 mM PIPES (pH 6.8), 100 mM NaCl, 300 mM sucrose, 3 mM MgCl2, 1 mM EDTA, 1 mM DTT, 0.5% (vol/vol) TritonX‐100, protease inhibitors (Roche)], 100 μl was removed for whole cell lysate and mixed with 100 μl SDS lysis buffer (1%SDS, 2% 2‐mercaptoethanol), and the remaining 100 μl was placed on ice for 10 min. Nuclei were then pelleted by centrifugation at 5,000 *g* for 5 min. The supernatant 100 μl cytosolic fraction was removed and mixed with 100 μl SDS lysis buffer. The nuclei were then washed in 100 μl CSKT buffer and centrifuged at 5,000 *g* for 5 min. The nuclei were then resuspended in 100 μl CSKT buffer followed by addition of 100 μl SDS lysis buffer to make the nuclear fraction. Samples were boiled for 10 min followed by vortexing for 30 s. Equal volumes of each fraction and whole cell lysate were separated on 4–12% NuPAGE gel (Thermo Fisher) with PAGEruler prestained protein markers (Thermo Fisher), transferred to Protran membrane (Amersham), and the blot was cut into three pieces based on prestained protein markers. The strip with proteins 70 kD and larger was incubated with mouse anti‐RNase L antibody mouse. The strip with proteins 70 to 25 kD was incubated with anti‐GAPDH‐HRP. The strip with proteins 25 kD and smaller was incubated with anti‐Histone H3 antibody. After incubation with appropriate HRP‐conjugated secondary antibodies, the blots were developed using SuperSignal West Dura substrate (Pierce). Images of blots obtained with ImageQuant LAS 4000 (GE Healthcare). Western blotting using anti‐RNase L antibody against DCP1‐RNase L fusion proteins was performed on whole cell lysates prepared as described above.

### Sequential immunofluorescence and FISH analysis following transfection with poly(I:C)

Cells were seeded on sterile coverslips in six‐well plates, grown to 40–60% confluence, then transfected with poly(I:C) HMW (InvivoGen: tlrl‐pic) using 3 μl of lipofectamine 2000 (Thermo Fisher Scientific) per 1 μg of poly(I:C) or mock transfected using lipofectamine alone for 4 to 5 h. Actinomycin D (Tocris), when used, was added to final concentration of 1 μg/ml at the same time as poly(I:C) addition. Sequential immunofluorescence and FISH analysis was performed following manufacturer’s protocol (https://biosearchassets.blob.core.windows.net/assets/bti_custom_stellaris_immunofluorescence_seq_protocol.pdf) with the following changes: 4% paraformaldehyde in 1XPBS was used for fixation, antibody incubations were performed in 0.1 ml 1% nuclease free BSA (Invitrogen) 1XPBS after blocking for 1 h in same solution, Dapi staining was performed in PBS following Wash Buffer B step, and coverslips were mounted using Prolong Glass (Invitrogen). FISH probes: human GAPDH with Quasar 670 dye (Stellaris SMF‐2019‐1), human MALAT1 with Quasar 670 dye (Stellaris SMF‐2046‐1), human snoRD3A probe GCGTTCTCTCCCTCTCACTCCCCAATA‐AlexaFluor647, and oligod(T)30‐Cy3 were purchased from IDT. ETS1 probe set was described in Yao *et al* (2019) and was labeled using 5‐propargylamino‐ddUTP‐ATTO633 (Axxora) and protocol adapted from Gaspar *et al* (2017).

### Microscopy and image analysis

Immunofluorescence and FISH with Dapi staining were imaged using either a widefield DeltaVision Elite Deconvolution microscope with an Olympus UPlanSApo 100X/1.40‐NA oil objective lens and a PCO Edge sCMOS camera with appropriate filters at room temperature using SoftWoRx Imaging software taking 15 Z planes at 0.2 μm/section per image or a Nikon Ti‐E spinning disk confocal microscope with a Nikon 100X/1.45‐NA oil objective lens and an Andor iXon Ultra 888 EMCCD camera (University of Colorado‐Boulder, BioFrontiers Advanced Light Microscopy Core). For images in figures, deconvolved widefield or confocal images were processed using ImageJ with Fiji package. Max projections of Z‐planes were performed, and brightness and contrast adjusted for each channel to best view results. Quantification of the number and volume of SGs, PBs, PMLs, MED1 foci, BRD4 foci, intermediate filaments and microtubules, RPF1 foci, SRRM2 foci, FBL foci, oligo(dT) signal, snoRD3A RNA, MALAT1 RNA, and ETS1 signal was performed using Imaris Image Analysis Software (Bitplane) (University of Colorado‐Boulder, BioFrontiers Advanced Light Microscopy Core) using confocal or non‐deconvolved widefield images. SRRM2 and SON partition coefficients were determined using Fiji plot profile to determine the fluorescent intensity of each protein in the nucleoplasm and in foci. All statistical tests were performed using Prism 9 software.

## Author contributions

**Carolyn J Decker:** Conceptualization; Data curation; Formal analysis; Investigation; Visualization; Methodology; Writing—original draft; Writing—review & editing. **James M Burke:** Conceptualization; Funding acquisition; Investigation; Writing—review & editing. **Patrick K Mulvaney:** Investigation; Writing—review & editing. **Roy Parker:** Conceptualization; Supervision; Funding acquisition; Writing—review & editing.

## Supporting information



Expanded View Figures PDF

Source Data for Expanded View

Source Data for Figure 1

Source Data for Figure 2

Source Data for Figure 3

Source Data for Figure 5

## Data Availability

Microscopy Image Files: BioStudies S‐BIAD359 (https://www.ebi.ac.uk/biostudies/studies/S‐BIAD359).
