# Supplementary material for: RNA is required for the integrity of multiple nuclear and cytoplasmic membrane‐less RNP granules
Source: EMBO J. 2022 Mar 31;41(9):e110137. doi: 10.15252/embj.2021110137 (PMC9058542; doi:10.15252/embj.2021110137)
Supplement: Supplementary file 1 — Expanded View Figures PDF [file EMBJ-41-e110137-s002.pdf]

Expanded View Figures

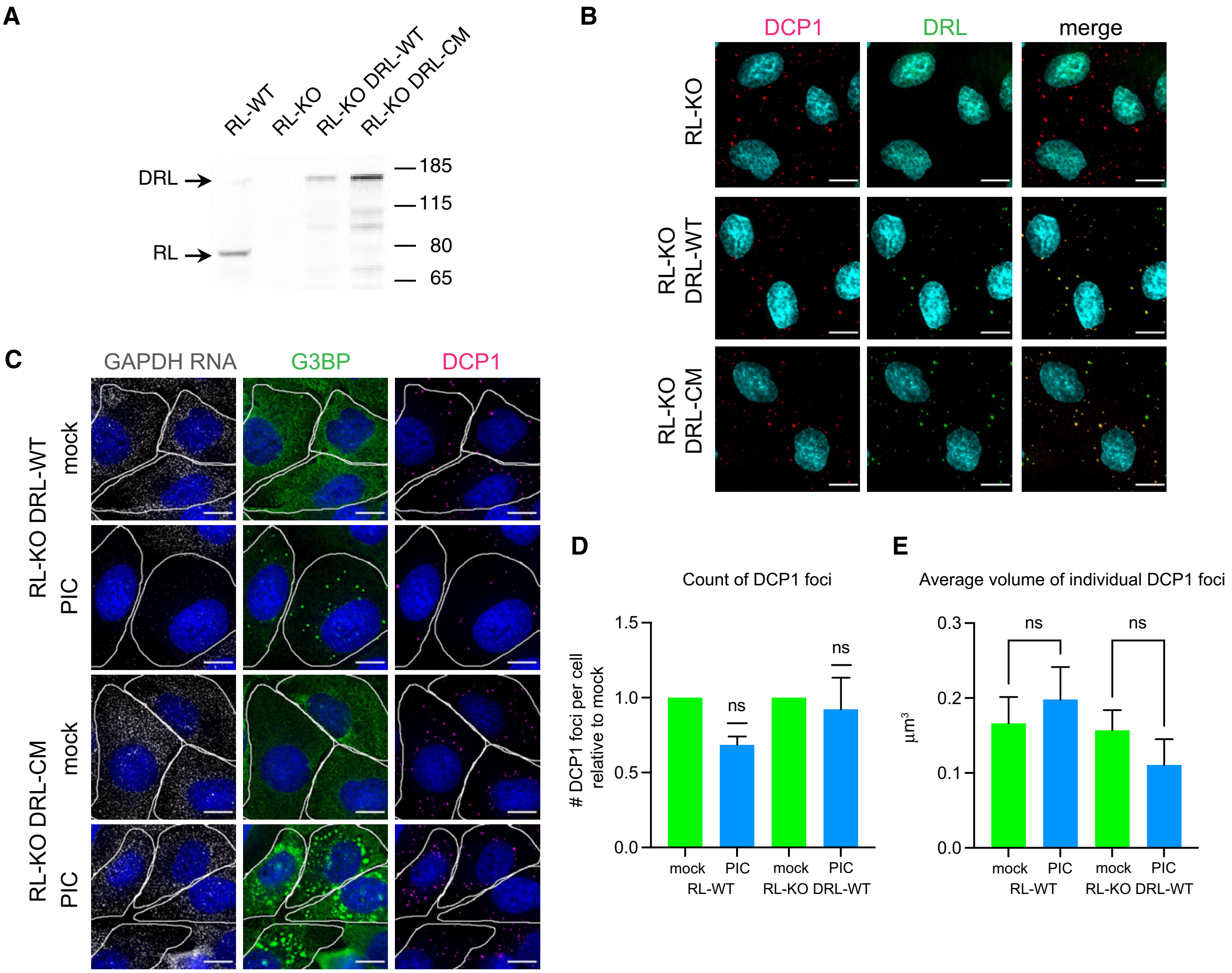

Figure EV1. Targeting RNase L to P-bodies did not increase the effect of RNase L activation on P-body number or volume.

A Full-length Dcp1 RNase L (DRL) fusion proteins are expressed. Western analysis using anti-RNase L antibody of whole cell lysates from A549 (RL-WT) cells, A549 RNase L knock out cells (RL-KO), and RL-KO cells transduced with lentiviral vectors containing either wild-type RNase L fused to DCP1a (RL-KO DRL-WT) or catalytic mutant RNase L-R667A fused to DCP1a (RL-KO DRL-CM). Arrows on left indicate migration of the endogenous RNase L (RL) and the DCP1 RNase L fusion proteins (DRL).

B DRL fusion proteins co-localize with P-bodies. IF analysis using anti-DCP1b antibody to detect P-bodies (DCP1) or anti-Flag antibody to detect Flag-tagged DCP1a-RNase L fusion proteins. Scale bar 10 microns.

C DRL-WT fusion protein is active. GAPDH smFISH and IF analysis using anti-G3BP antibody (G3BP) and anti-Dcp1b antibody (DCP1) in A549 RL-KO cells with DRL-WT or DRL-CM fusion proteins either mock transfected (mock) or transfected with poly(I:C) (PIC) for 5 h. Scale bar 10 microns.

D Number of DCP1 foci per cell in mock- and PIC-treated RL-WT and RL-KO cells expressing DRL-WT relative to the number in mock-treated cells. Wilcoxon Signed Rank test, ns, non-significant

E Average volume of individual DCP1 foci in mock- and PIC-treated cells. One-way ANOVA with Sidak's multiple comparisons test.

Data information: (D, E) Bar graphs show mean + SD for $N = 4$ independent experiments. ns, non-significant.
Source data are available online for this figure.

 © 2022 The Authors

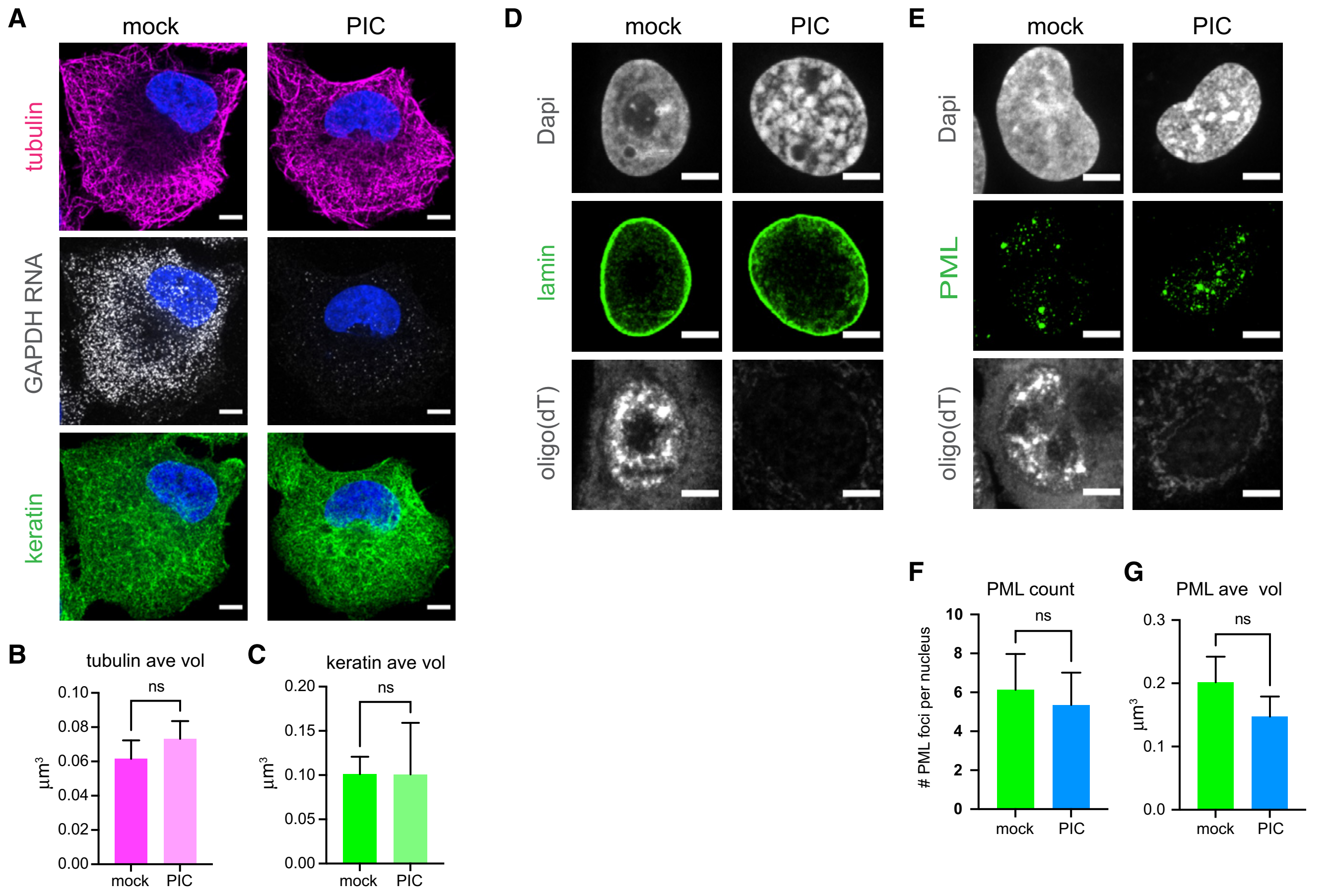

Figure EV2. Degradation of RNA in the cytoplasm does not alter microtubule or intermediate filament networks and nuclear RNA degradation does not alter the integrity of the nuclear lamin or PML bodies.

A GAPDH smFISH and IF analysis of microtubules (anti-alpha tubulin antibody) or intermediate filaments (anti-pan keratin antibody) in A549 cells mock treated or treated with poly(I:C) for 5 h.

B Graph of average volume of tubulin structures in mock- and PIC-treated cells with reduced GAPDH RNA.

C Graph of average volume of keratin structures in mock- and PIC-treated cells with reduced GAPDH RNA.

D, E A549 cells expressing nuclear-localized wild-type RNase L mock or poly(I:C) treated for 5 h. (D) Oligo(dT) FISH and IF analysis of nuclear lamin (anti-lamin A antibody). (E) Oligo(dT) FISH and IF analysis of PML bodies (anti-PML antibody).

F Graph of the number of PML foci in mock- and PIC-treated cells with reduced oligo(dT) signal.

G Graph of average volume of PML foci in mock- and PIC-treated cells with reduced oligo(dT) signal.

Data information: All images scale bar 5 micron. (B, C) Mean and SEM of four experiments with at least 8 cells per condition per experiment analyzed. (F, G) Mean and SEM of three experiments with at least 10 cells per condition per experiment analyzed. All graphs unpaired two-tailed *t*-test, ns, non-significant.
Source data are available online for this figure.

© 2022 The Authors

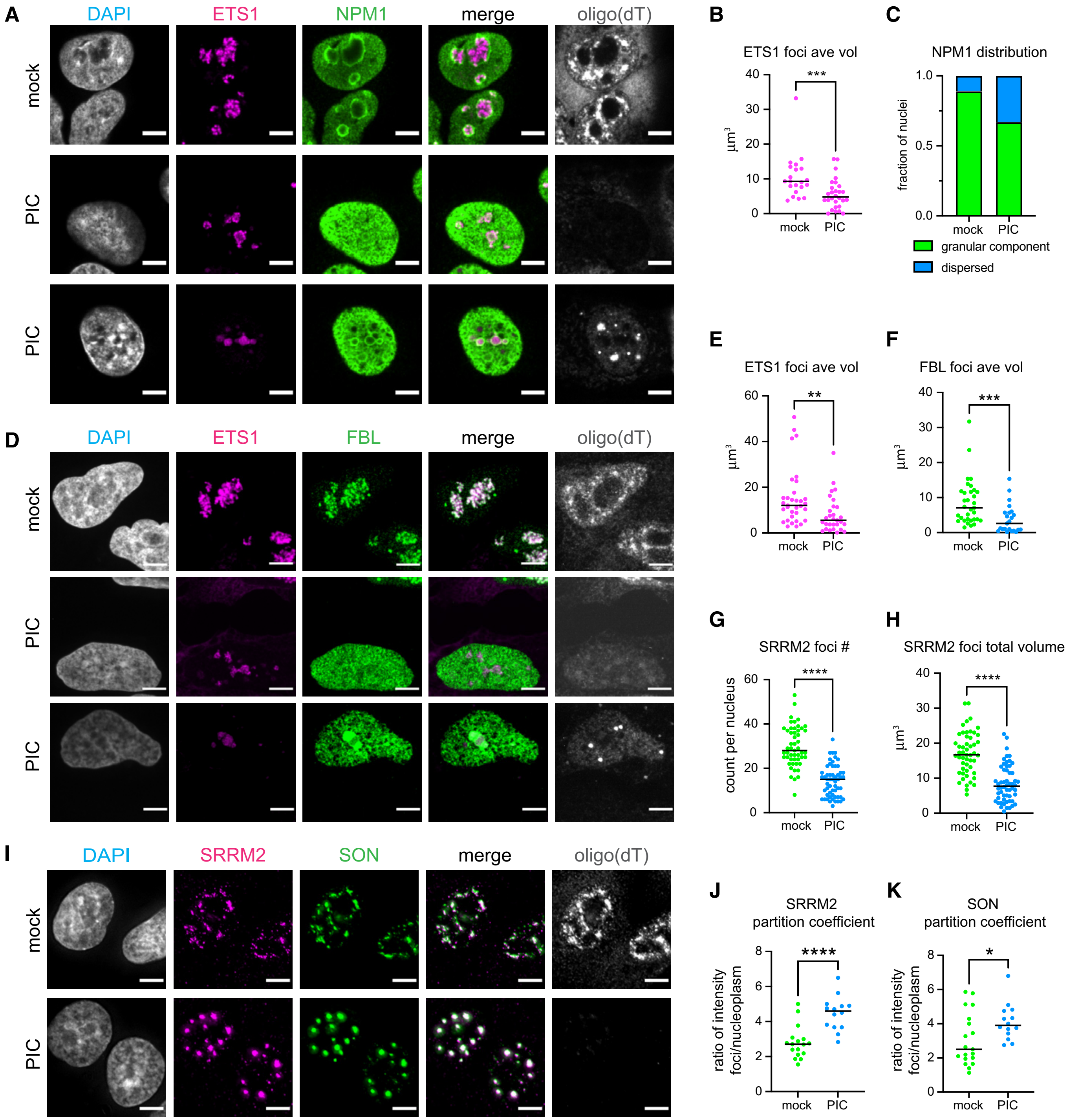

Figure EV3.

 © 2022 The Authors

Figure EV3. Effect of activation of RNase L in the nucleus on precursor rRNA and nucleolar proteins and on nuclear speckles.

Analysis of A549 cells expressing nuclear-localized wild-type RNase L mock or poly(I:C) treated for 5 h.

A ETS1 and oligo(dT) FISH and IF analysis of NPM1 protein.
B Graph of the mean and individual values for the average volume of ETS1 FISH signal in nuclei. ***P-value = 0.0009. Mock 21 nuclei. PIC 28 nuclei.
C Fraction of nuclei with NPM1 protein enriched in ring structures of any size classified as granular component assemblies or dispersed in nucleoplasm. Mock 21 nuclei. PIC 24 nuclei in which ETS1 signal was lower than the median ave vol in mock-treated cells.
D ETS1 and oligo(dT) FISH and IF analysis of FBL protein.
E Graph of the mean and individual values for the average volume of ETS1 FISH signal in nuclei. Mock 33 nuclei. PIC 29 nuclei. **P-value = 0.0025.
F Graph of the mean and individual values for the average volume of fibrillarin (FBL) foci in nuclei. Mock 33 nuclei. PIC 23 nuclei in which ETS1 signal was lower than the median ave vol in mock-treated cells. ***P-value = 0.0008.
G Graph of the mean and individual value for the number of SRRM2 foci per nucleus. ****P-value $\leq$ 0.0001 Mock 51 nuclei. PIC 56 nuclei.
H Graph of the mean and individual value for the total volume of SRRM2 foci divided by the nuclear volume of individual nuclei. ****P-value $\leq$ 0.0001 Mock 51 nuclei. PIC 56 nuclei.
I Oligo(dT) FISH and IF analysis of two nuclear speckle proteins, SRRM2 and SON.
J Graph of the median and individual value of the ratio of SRRM2 fluorescence intensity in foci compared to in the nucleoplasm. ****P-value $\leq$ 0.0001 Mock 17 foci in 5 nuclei. PIC 14 foci in 5 nuclei.
K Graph of the median and individual value of the ratio of SON fluorescence intensity in foci compared to in the nucleoplasm. *P-value = 0.034 Mock 19 foci in 5 nuclei. PIC 14 foci in 5 nuclei.

Data information: Scale bar 5 micron. All graphs with significance test Mann–Whitney two-tailed test.
Source data are available online for this figure.

© 2022 The Authors

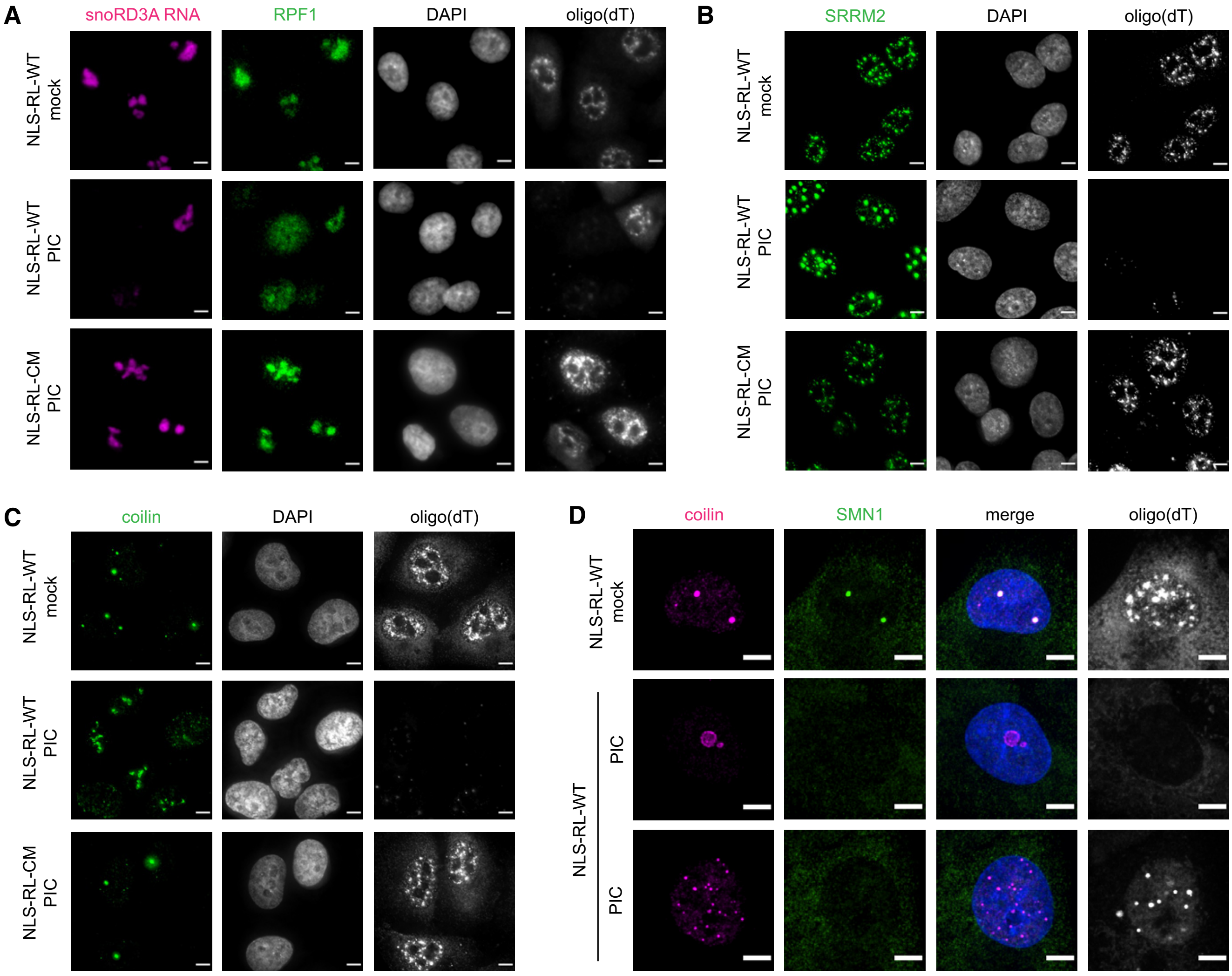

Figure EV4. Changes in nuclear RNA granule morphology is dependent on loss of nuclear RNA and SMN1 disperses into nucleoplasm in response to nuclear RNA degradation.

IF analysis of nuclear RNA granule proteins in A549 cells expressing nuclear-localized wild-type RNase L or catalytic mutant RNase L-R667A mock transfected or treated with poly(I:C) for 5 h.

A FISH analysis to detect nucleolar-localized snoRD3A RNA and poly(A)+ RNA. IF analysis of nucleolar protein RPF1.
B Oligo(dT) FISH and IF analysis of nuclear speckle protein SRRM2 with sc35 antibody.
C Oligo(dT) FISH and IF analysis of Cajal body protein coilin.
D Oligo(dT) FISH and IF analysis of Cajal body protein coilin and SMN1.

Data information: Scale bar 5 microns.

© 2022 The Authors

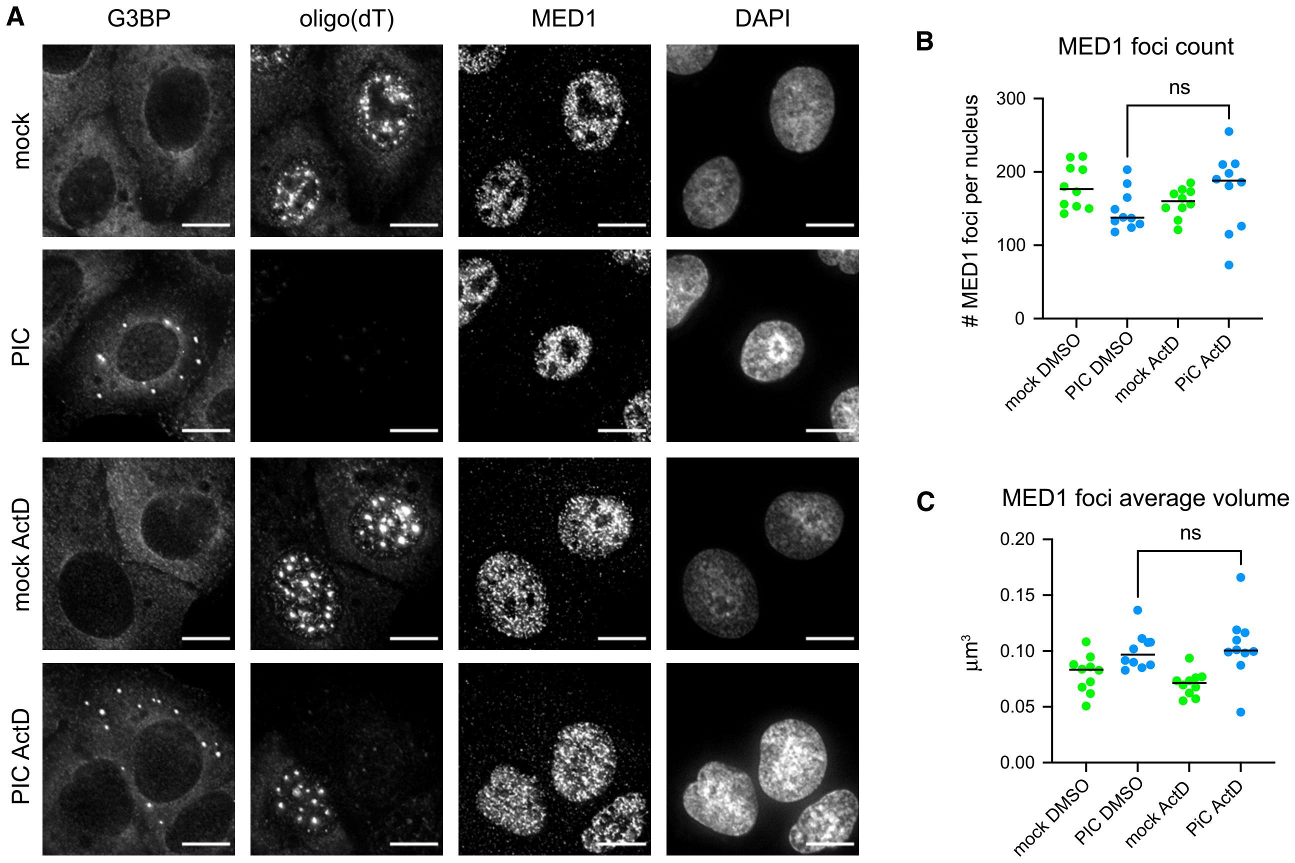

Figure EV5. Inhibition of transcription in combination with nuclear RNA degradation did not affect the number or volume of MED1 foci.

A IF analysis of super-enhancer condensate protein MED1 in A549 cells expressing nuclear-localized wild-type RNase L mock transfected or treated with poly(I:C) with DMSO or 1 µg/ml Actinomycin D (ActD) for 5 h. IF against G3BP used to monitor cells responding to poly(I:C), FISH analysis for poly(A)+ RNA used to monitor nuclear RNA degradation and transcription inhibition. Scale bar 5 microns.

B Number of MED1 foci per cell in mock-, PIC-, ActD-, or PIC and ActD-treated cells.

C Average volume of individual MED1 foci per cell in mock-, PIC-, ActD-, or PIC and ActD-treated cells.

Data information: (B, C) Graphs depict the mean and individual values for each cell. At least 10 cells in each condition were counted. Unpaired, two-tailed *t*-test, ns non-significant.

Source data are available online for this figure.

© 2022 The Authors
