# Supplementary material for: RNA is required for the integrity of multiple nuclear and cytoplasmic membrane‐less RNP granules
Source: EMBO J. 2022 Mar 31;41(9):e110137. doi: 10.15252/embj.2021110137 (PMC9058542; doi:10.15252/embj.2021110137)

DV image 020621 sum projection nondeconvolved images

mock

4A01 ch2=SRRM2

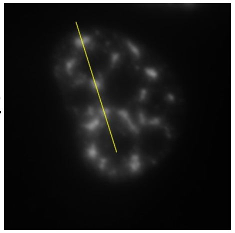

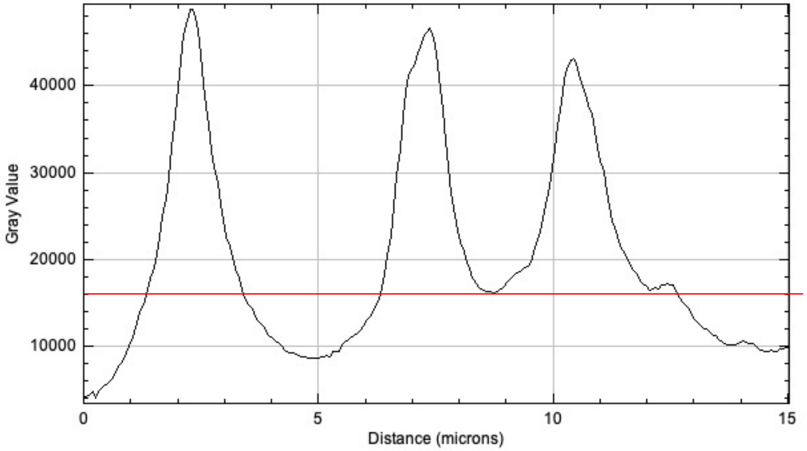

4A01 ch0=SON

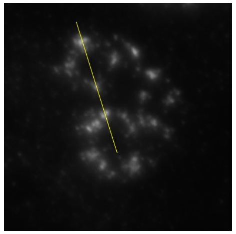

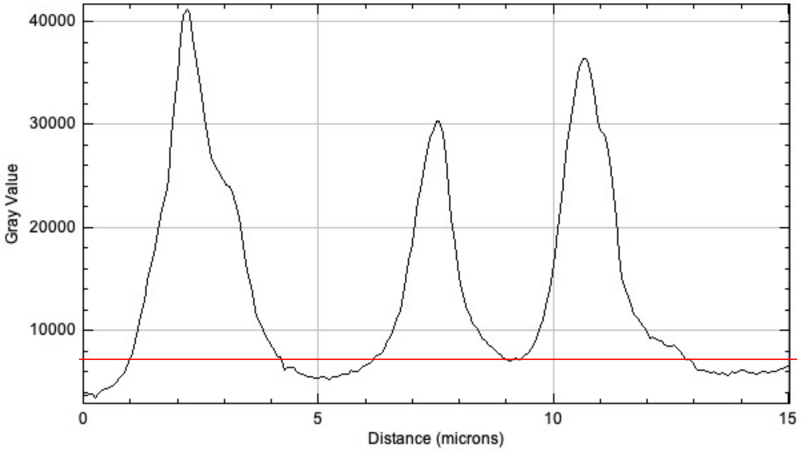

DV image 020621 sum projection nondeconvolved images

mock

4A02 ch2=SRRM2

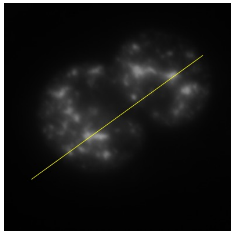

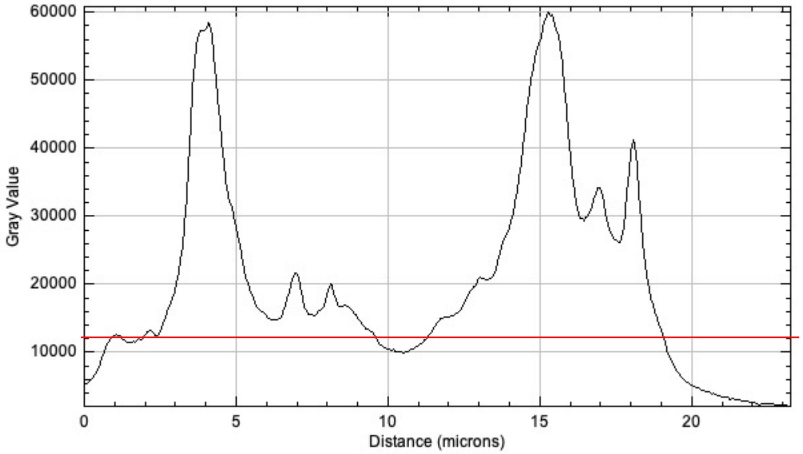

4A02 ch0=SON

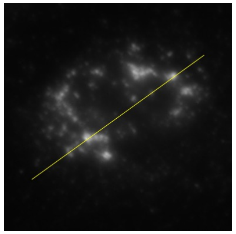

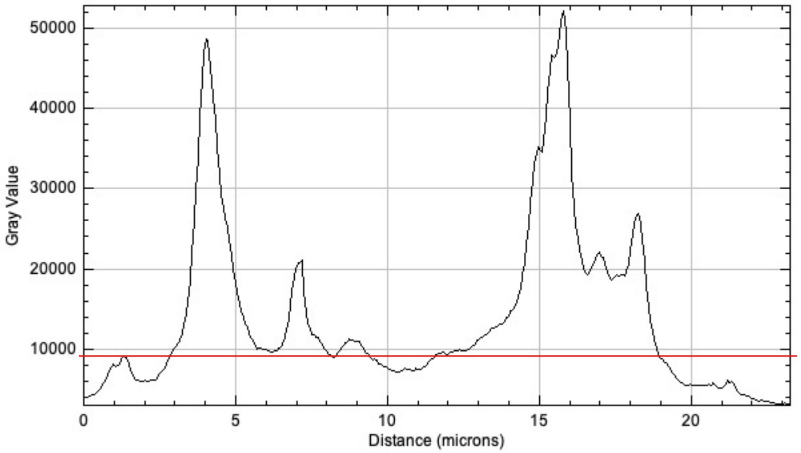

DV image 020621 sum projection nondeconvolved images

mock

4A03 ch2=SRRM2

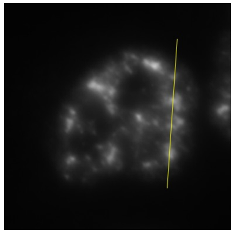

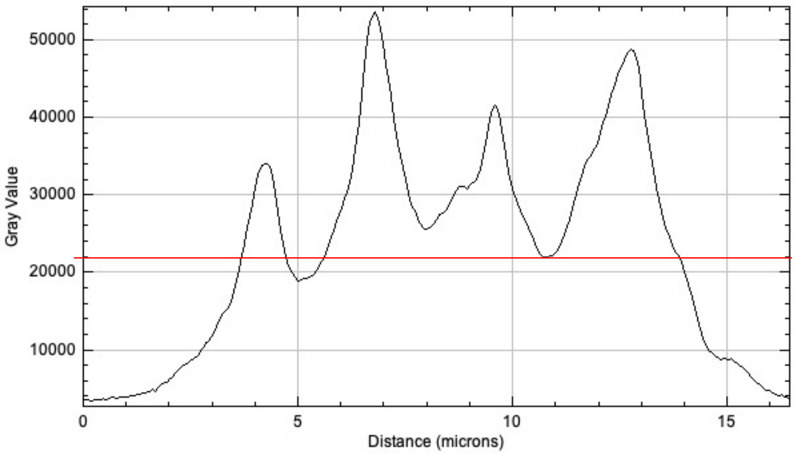

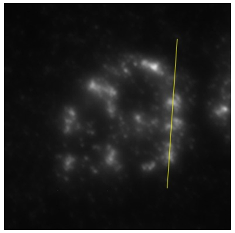

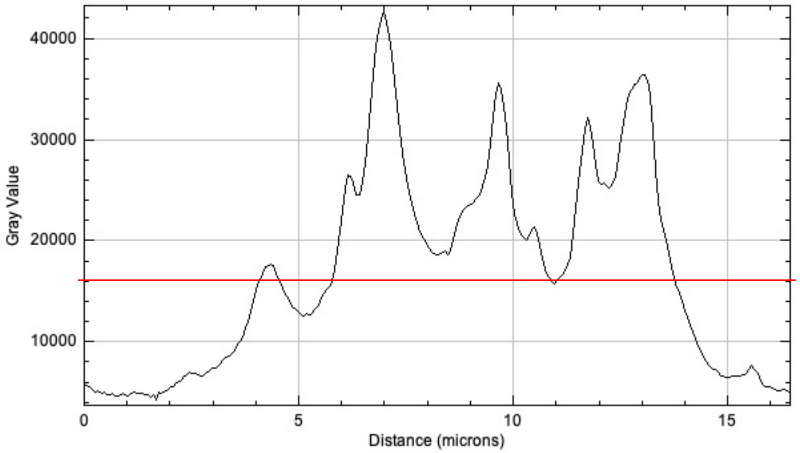

4A03 ch0=SON

DV image 020621 sum projection nondeconvolved images

mock

4A04 ch2=SRRM2

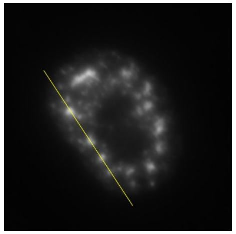

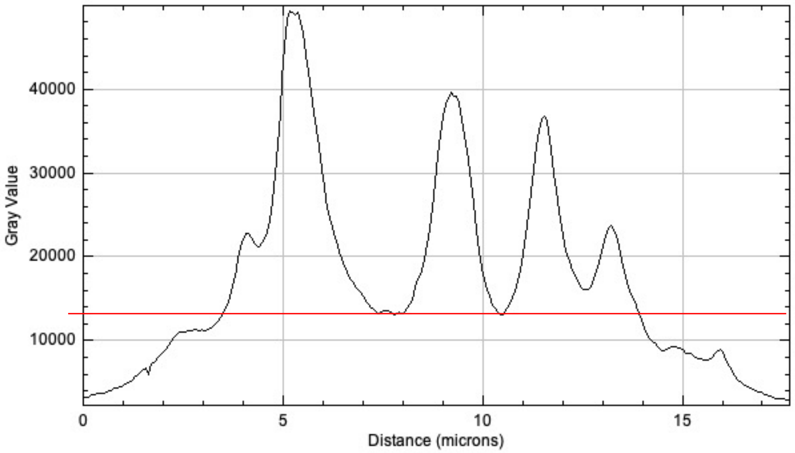

4A04 ch0=SON

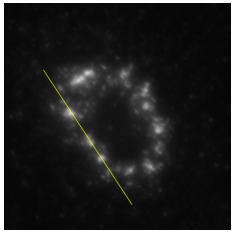

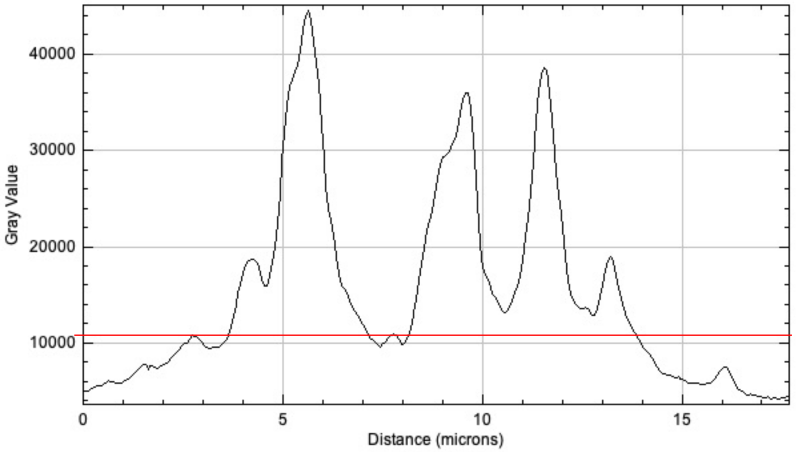

DV image 020621 sum projection nondeconvolved images

mock

4A04 second nucleus ch2=SRRM2

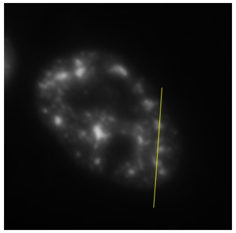

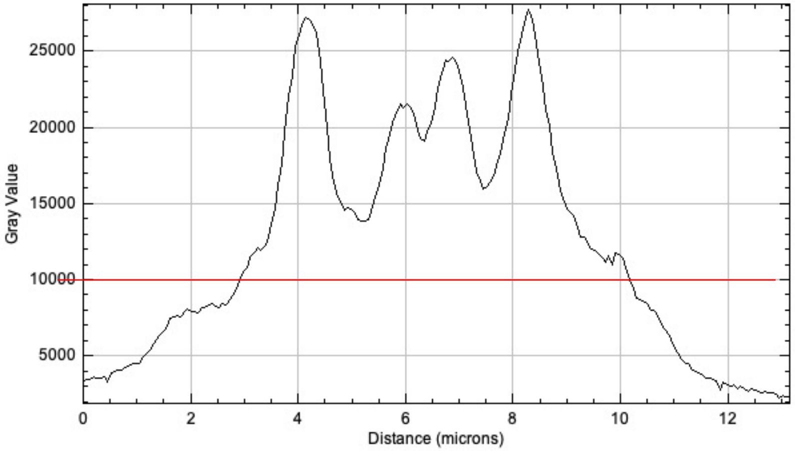

4A04 second nucleus ch0=SON

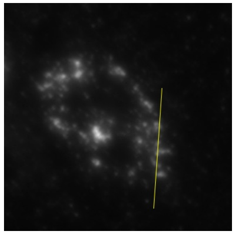

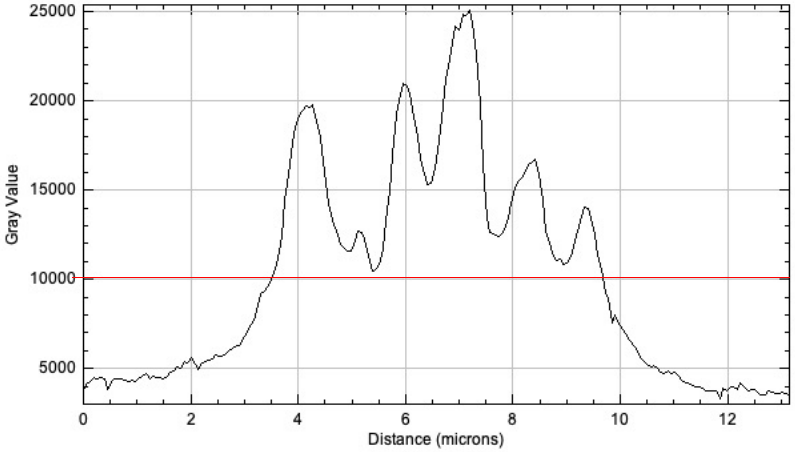

DV image 020621 sum projection nondeconvolved images

PIC

4B01 ch2=SRRM2

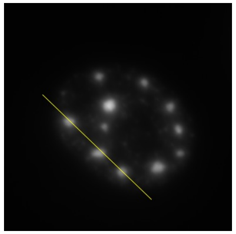

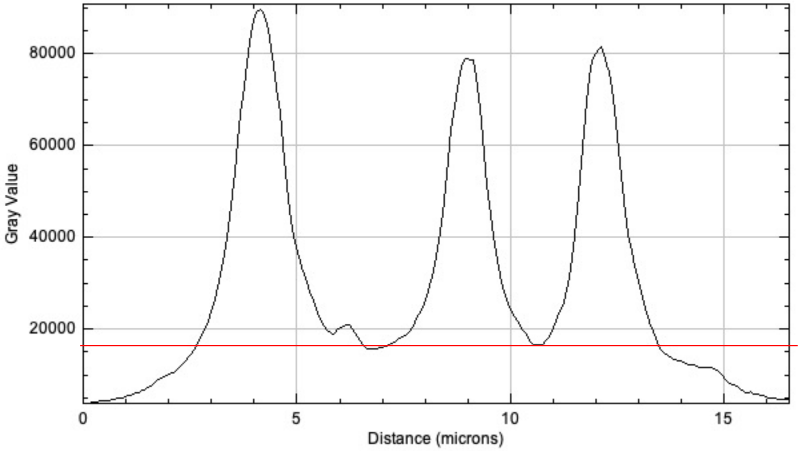

4B01 ch0=SON

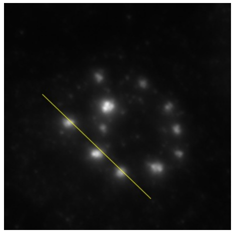

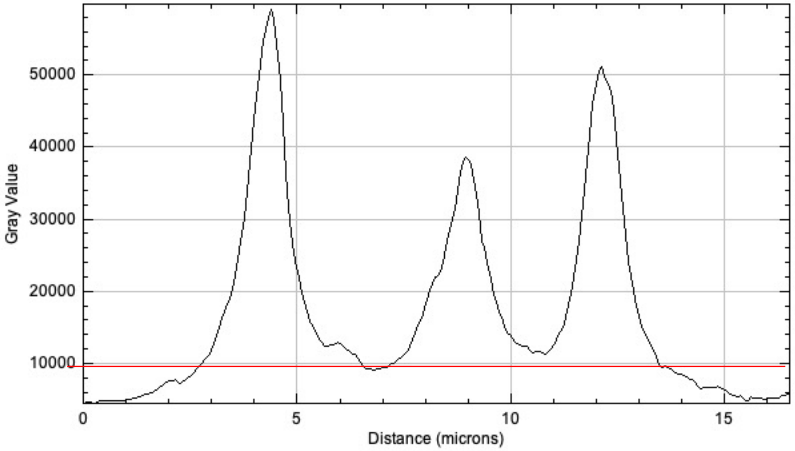

DV image 020621 sum projection nondeconvolved images

PIC

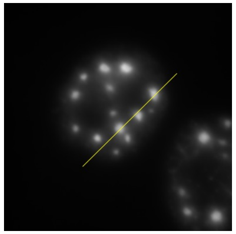

4B01 ch2=SRRM2
second nucleus

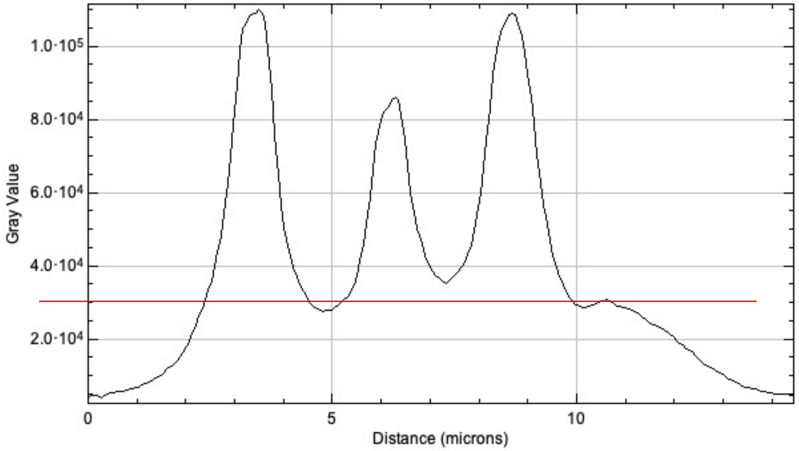

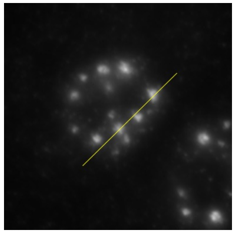

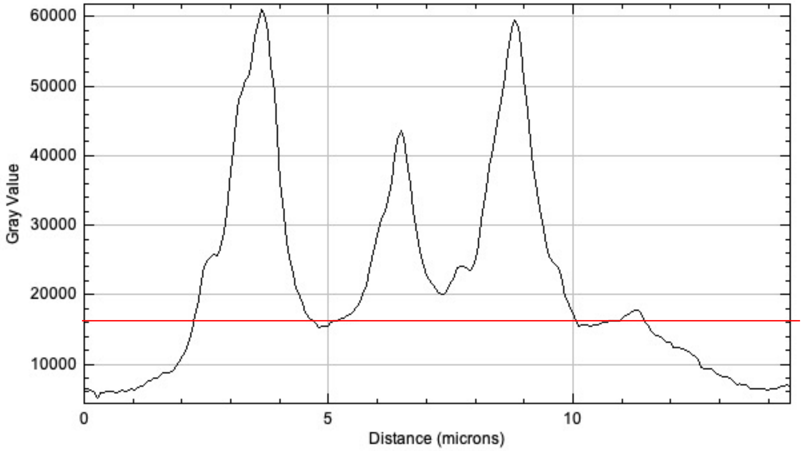

4B01 ch0=SON
second nucleus

DV image 020621 sum projection nondeconvolved images

PIC

4B03 ch2=SRRM2

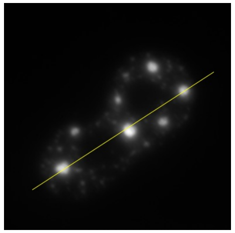

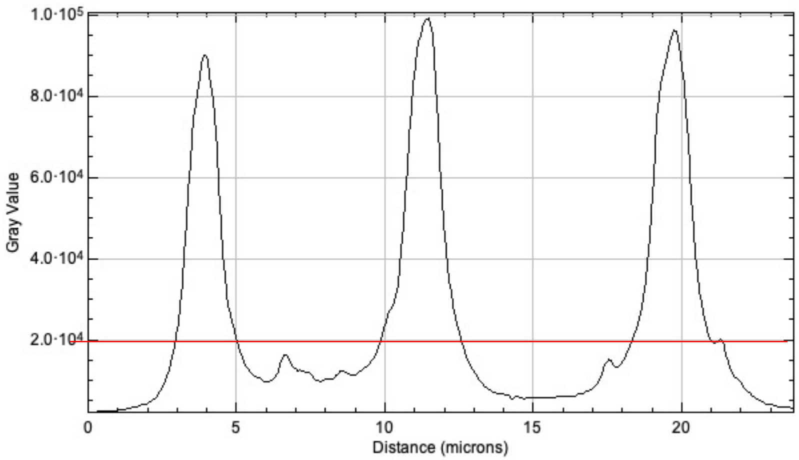

4B03 ch0=SON

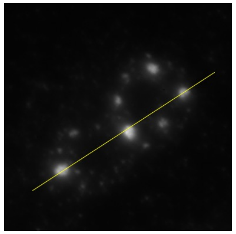

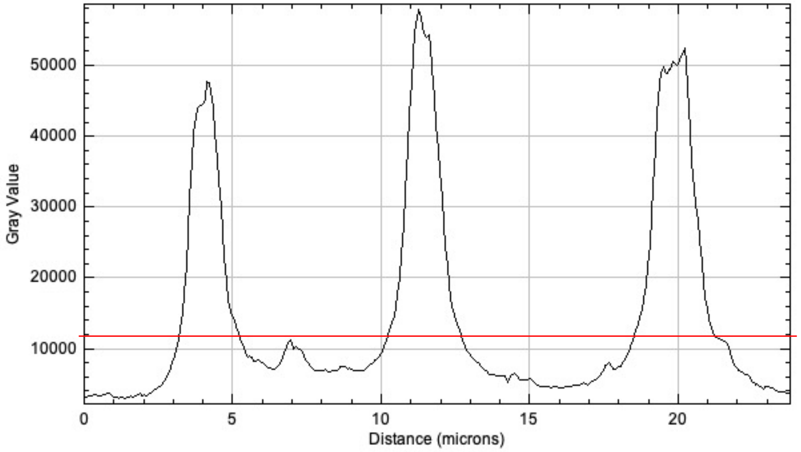

DV image 020621 sum projection nondeconvolved images

PIC

4B04 ch2=SRRM2

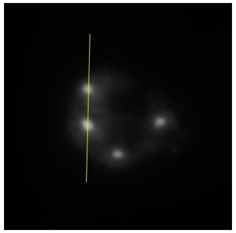

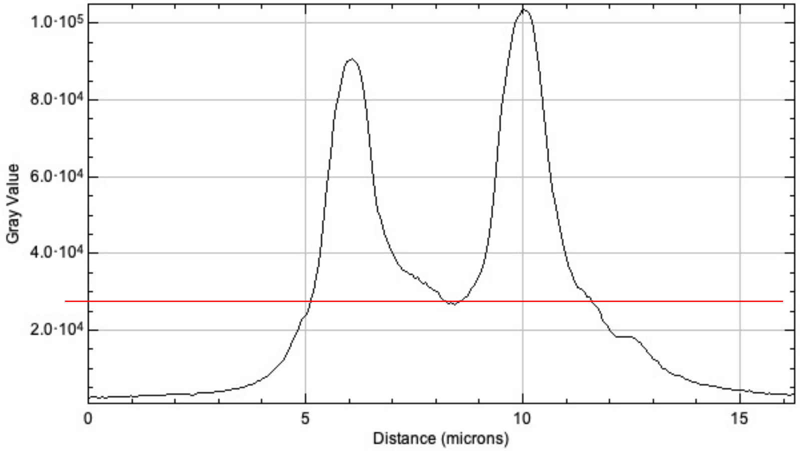

4B04 ch0=SON

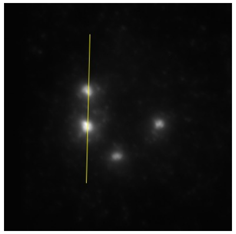

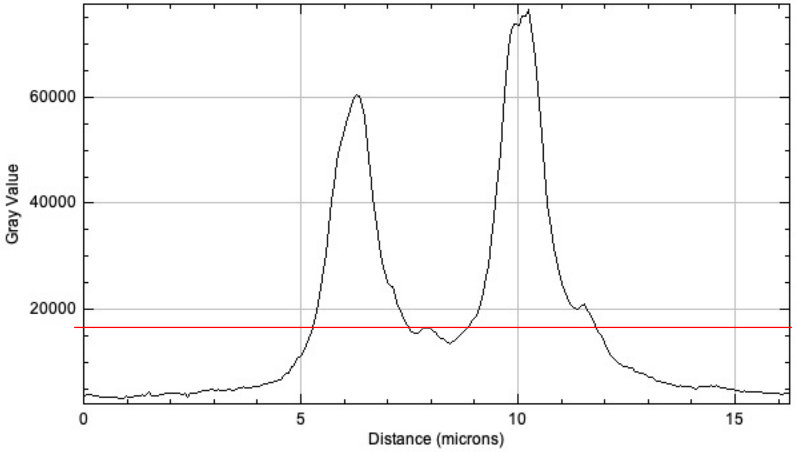

Supplement: Supplementary file 2 — Source Data for Expanded View [file EMBJ-41-e110137-s007.zip › EMBOJ-2021-110137_Source_Data_EVfigures/EMBOJ-2021-110137_SourceDataforFigEV3/FigEV3J/FigEV3_JK_partitioncoeff_plotprofiles.pdf]
