# Supplementary figures and images for: RNA is required for the integrity of multiple nuclear and cytoplasmic membrane‐less RNP granules
Source: EMBO J. 2022 Mar 31;41(9):e110137. doi: 10.15252/embj.2021110137 (PMC9058542; doi:10.15252/embj.2021110137)

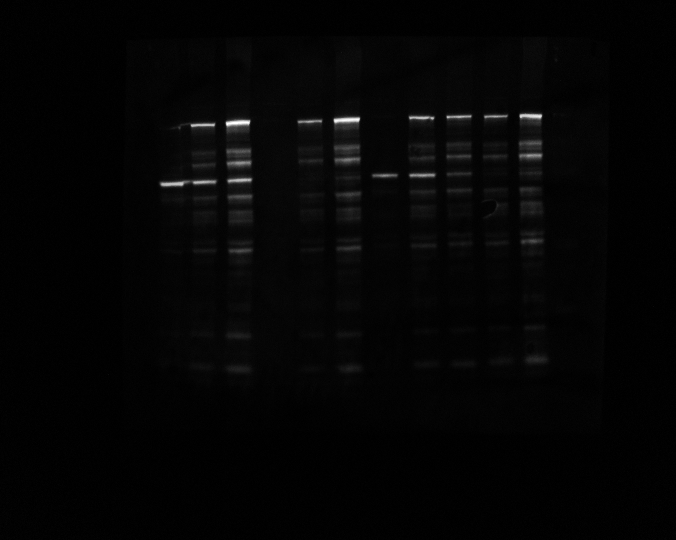

Supplement: Supplementary file 2 — Source Data for Expanded View [file EMBJ-41-e110137-s007.zip › EMBOJ-2021-110137_Source_Data_EVfigures/EMBOJ-2021-110137_SourceDataforFigEV1/FigEV1A/FigEV1A_RNaseL_western_chemiluminescence.tif]

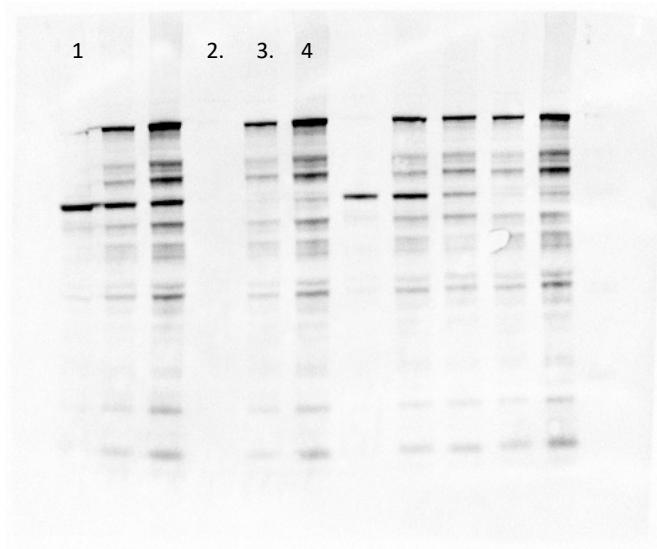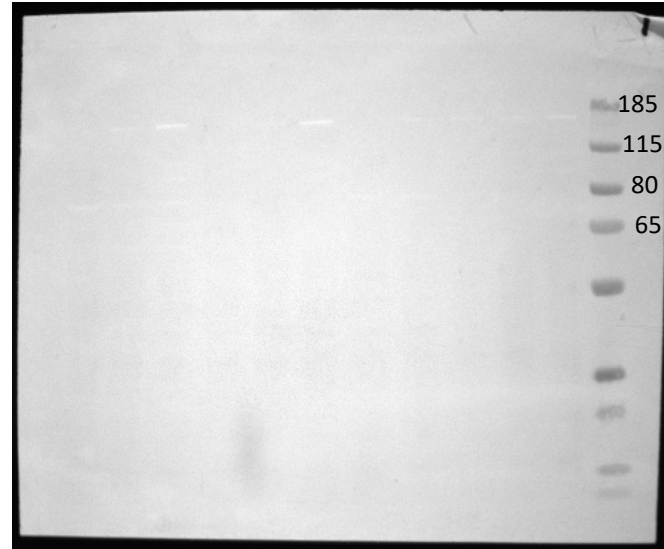

1. RL-WT A549
2. RL-KO A549
3. RL-KO A549 with Dcp1 fused to RL-WT
4. RL-KO 549 with Dcp1 fused to RL-CM

Supplement: Supplementary file 2 — Source Data for Expanded View [file EMBJ-41-e110137-s007.zip › EMBOJ-2021-110137_Source_Data_EVfigures/EMBOJ-2021-110137_SourceDataforFigEV1/FigEV1A/FigEV1A_RNaseL_western_laneinformation.pdf]

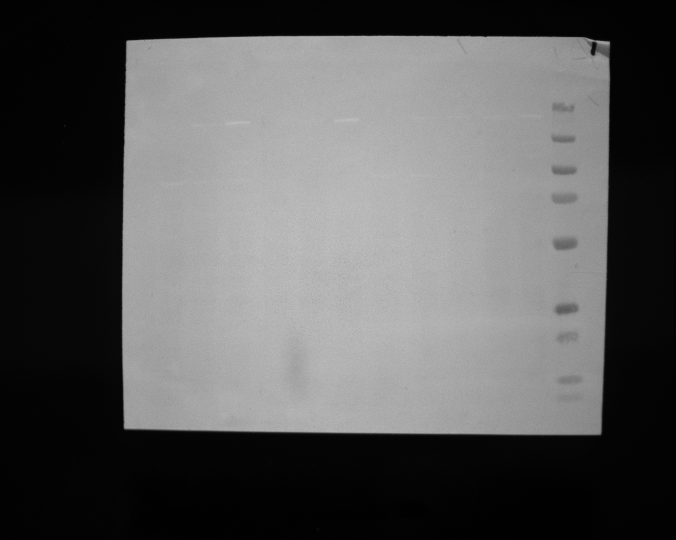

Supplement: Supplementary file 2 — Source Data for Expanded View [file EMBJ-41-e110137-s007.zip › EMBOJ-2021-110137_Source_Data_EVfigures/EMBOJ-2021-110137_SourceDataforFigEV1/FigEV1A/FigEV1A_RNaseL_western_membran.tif]

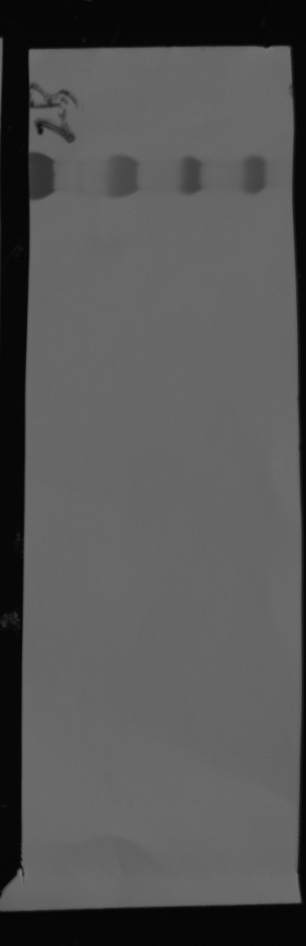

Supplement: Supplementary file 4 — Source Data for Figure 2 [file EMBJ-41-e110137-s001.zip › EMBOJ-2021-110137_Source_Data_Fig2/Fig2A/Fig2A_GAPDH_blot.tif]

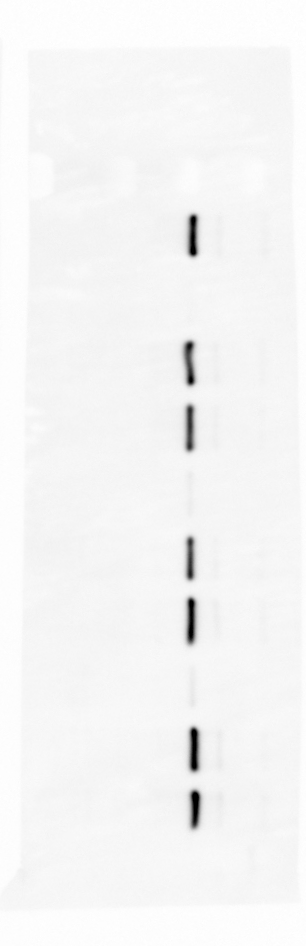

Supplement: Supplementary file 4 — Source Data for Figure 2 [file EMBJ-41-e110137-s001.zip › EMBOJ-2021-110137_Source_Data_Fig2/Fig2A/Fig2A_GAPDH_western.tif]

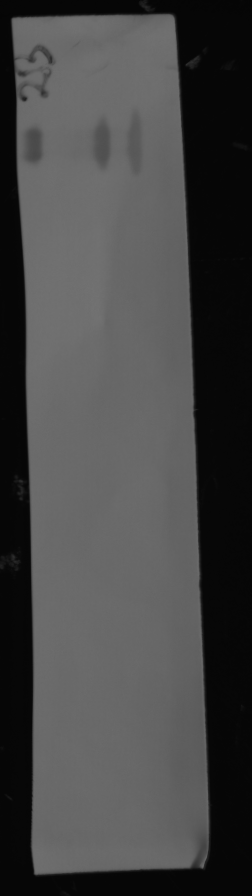

Supplement: Supplementary file 4 — Source Data for Figure 2 [file EMBJ-41-e110137-s001.zip › EMBOJ-2021-110137_Source_Data_Fig2/Fig2A/Fig2A_HistoneH3_blot.tif]

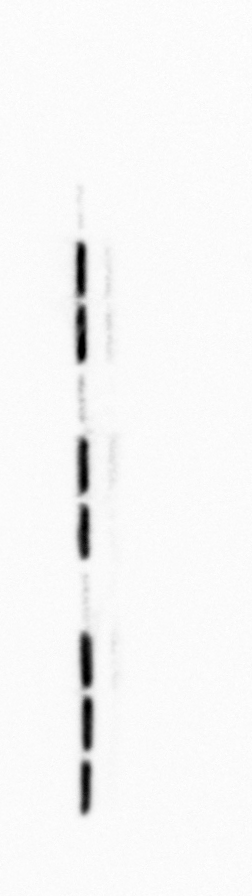

Supplement: Supplementary file 4 — Source Data for Figure 2 [file EMBJ-41-e110137-s001.zip › EMBOJ-2021-110137_Source_Data_Fig2/Fig2A/Fig2A_HistoneH3_western.tif]

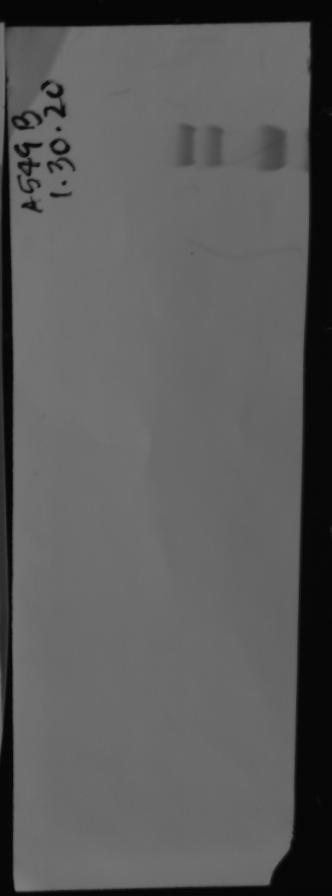

Supplement: Supplementary file 4 — Source Data for Figure 2 [file EMBJ-41-e110137-s001.zip › EMBOJ-2021-110137_Source_Data_Fig2/Fig2A/Fig2A_RNaseLlongexp_blot.tif]

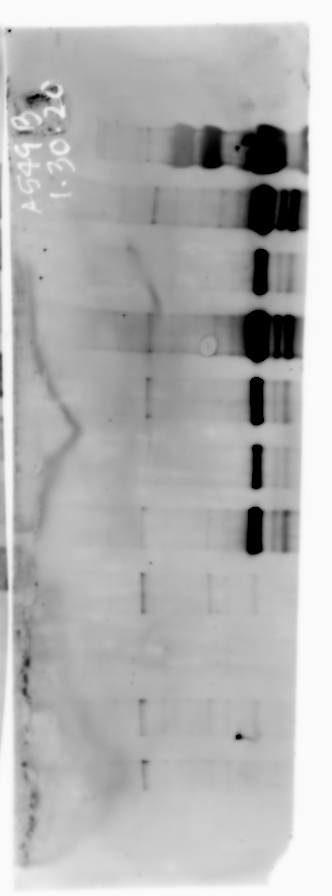

Supplement: Supplementary file 4 — Source Data for Figure 2 [file EMBJ-41-e110137-s001.zip › EMBOJ-2021-110137_Source_Data_Fig2/Fig2A/Fig2A_RNaseLlongexp_western.tif]

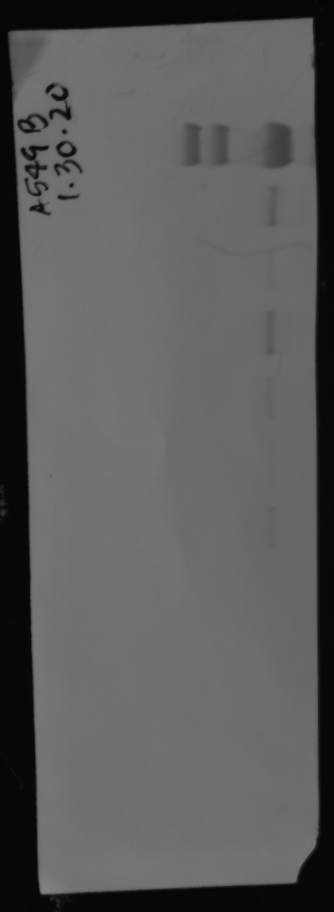

Supplement: Supplementary file 4 — Source Data for Figure 2 [file EMBJ-41-e110137-s001.zip › EMBOJ-2021-110137_Source_Data_Fig2/Fig2A/Fig2A_RNaseLshortexp_blot.tif]

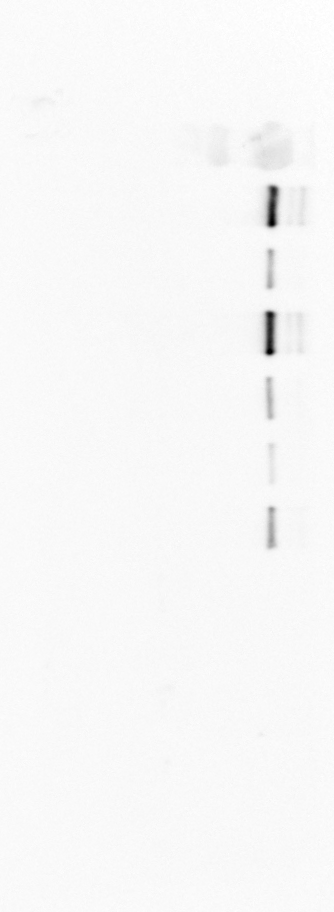

Supplement: Supplementary file 4 — Source Data for Figure 2 [file EMBJ-41-e110137-s001.zip › EMBOJ-2021-110137_Source_Data_Fig2/Fig2A/Fig2A_RNaseLshortexp_western.tif]
