# Supplementary material for: RNA is required for the integrity of multiple nuclear and cytoplasmic membrane‐less RNP granules
Source: EMBO J. 2022 Mar 31;41(9):e110137. doi: 10.15252/embj.2021110137 (PMC9058542; doi:10.15252/embj.2021110137)
Supplement: Supplementary file 7 [file EMBJ-41-e110137-s003.pdf]

THE EMBO JOURNAL

RNA is required for the integrity of multiple nuclear and cytoplasmic membrane-less RNP granules.

Carolyn Decker, James Burke, Patrick Mulvaney, and Roy Parker
DOI: 10.15252/embj.2021110137

Corresponding author(s): Roy Parker (roy.parker@Colorado.EDU)

Review Timeline:

Transfer from Review Commons:	6th Nov 21
Editorial Decision:	9th Nov 21
Revision Received:	28th Jan 22
Editorial Decision:	21st Feb 22
Revision Received:	3rd Mar 22
Accepted:	14th Mar 22

Editor: Stefanie Boehm

Transaction Report: This manuscript was transferred to The EMBO Journal following peer review at Review Commons.

Review COMMONS

(Note: With the exception of the correction of typographical or spelling errors that could be a source of ambiguity, letters and reports are not edited. Depending on transfer agreements, referee reports obtained elsewhere may or may not be included in this compilation. Referee reports are anonymous unless the Referee chooses to sign their reports.)

1st Editorial Decision 9th Nov 2021

Thank you for transferring your Review Commons manuscript to The EMBO Journal. I have now read your manuscript and response, as well as discussed everything with the other members of the editorial team. In light of the referees' positive comments and your revision plan, we would like to invite you to prepare and submit a revised manuscript.

As outlined in your revision plan, it will be important to address the reviewer's concerns experimentally or by revising the manuscript and to provide a careful response to each of their comments when submitting the revised manuscript. In particular, the characteristic features for the different responses of granules to RNA degradation should be further defined in the revised manuscript (ref #1- point 2; ref #3- point 4, 5, 6). In addition, in line with referee #1's point 6, we find that the discussion on the functional implications for the RNA-dependence vs. independence of granules should be expanded. When preparing the revised manuscript, please also refer to the journal's guidelines for submission of revisions (https://www.embopress.org/page/journal/14602075/authorguide#submissionofrevisions), at this stage the manuscript now has to adhere to the EMBO Journal formatting guidelines.

Transferred Referee reports

Review #1 -

Evidence, reproducibility and clarity (Required)

In the paper entitled. "RNA is required for the maintenance of multiple cytoplasmic and nuclear membrane-less organelles" Decker et al set out to test the rolw of RNA in maintaining the integrity of a variety of biomolecular condensates. To do this, they assess how multiple different assemblies in the cytoplasm and nucleus retain their structural integrity following RNAseL activation. They identified many condensates which are solubilized and have protein components redistributed following RNAse L activation and presumably subsequent RNA digestion. These experiments largely concur with previous findings from RNAseA treatment. The implication is that RNA rather than protein is the essential organizing component for most tested condensates. The manuscript is well written, and the data are convincing. It is my judgement that this is worthy of publication following a few additional experiments/clarifications.

1. The authors identify condensates which are sensitive to or refractory to RNAaseL. It would be good if the authors more conclusively eliminate the possibility that remaining condensates contain specific residual antiviral RNAs and this is the reason why these condensates remain intact. Are any of these condensates enriched in anti-viral RNAs like IFNbeta following polyIC treatment by FISH, for example (PMID: 31494035)?
2. Is there a particular protein feature, charge, IDR-types etc. which is common to solubilized versus not solubilized groups? What about dissolved and novel formed assemblies? A simple table comparing protein features in the three groups would suffice, with particular emphasis on RNA binding domains PMID: 32243832 and intrinsic disordered regions PMID: 24773235.
3. Demonstrate that the RNAseL treatment is reversible (i.e. withdraw polyIC, particularly for a protein that ends up in a novel assembly) or remove the word maintenance from the title.
4. Control for RNA-dependence of the activity. Try to dissolve a non-RNA dependent/enriched condensate with RNAseL. SPOP mutations (PMID: 30244836) might be interesting as both SPOP and RNAseL loss of function mutations (PMID: 11799394) are associated with prostate cancer.
5. A caveat is that certain regions of condensates enriched in RNA may not be accessible to RNAseL protein. A way to address this might be to attempt to directly target the enzyme to a compartment that is deemed refractory to the activity (and inferred to not require RNA) via an inducible systsem (ie FKBP12/FK506)
6. Overall, this paper would be greatly enhanced by including a more extensive discussion on the basic biological implications for these findings. Why are some condensates RNA dependent? What function(s) are common to these condensates? How does disruption of this lead to disease?

3. Significance:

Significance (Required)

This work addresses the neglected role of RNA in structuring condensates throughout the cell. Despite the prevalence of RNA in many condensates and the enrichment of RNA-binding proteins in condensates, there is still a highly limited understanding of the structural roles RNA plays in their assembly s most work has been protein/IDR-centric. This work seeks to systematically assess the RNA-dependence of the assemblies.

Review #2 -

Evidence, reproducibility and clarity (Required)

The manuscript by Decker et al. "RNA is required for the maintenance of multiple cytoplasmic and nuclear membrane-less organelles" investigates the structural role of RNA in membraneless organelles. The authors show that degradation of RNA in transient or constitutive membraneless organelles results in the altered formation and structure of many but not all organelles studied. The main assay is the activation of RNAseL activity by dsRNA which then destroys mRNA in the cell. The collected data leads the authors to highlight the possible roles of RNA in membraneless organelle formation and categorize the organelles: some relying more on the RNA-RNA interactions while others on protein-RNA or protein-protein interactions. The manuscript is well written and the data is sound.

Major comments:

The authors study the maintenance of organelles by RNA. For the transient ones, like stress granules (SG), it would be very interesting to see the formation/clearance kinetics with and without RNA. Also maybe using something other than dsRNA to trigger the formation. The idea being - if RNA is needed for SG maintenance, then the clearance kinetics with RNA would differ from that of the depleted RNA.

The experiments were done in cells. It is known that core components of the organelles can form granule like structures in vitro without RNA. If it is possible to show that RNA presence improves the integrity in vitro, that would support the authors claim. For example studying SG maintenance with and without RNAseL using the previously developed SG extraction protocol.

Minor comments:

In the Figure 1a, it is not clear if the smaller granules are different from SGs as mentioned in the text, maybe using additional markers can make it clearer.
Figure 3 and 4 requires quantification.

3. Significance:

Significance (Required)

This is a solid paper that advances our understanding of membraneless organelle formation and dynamics. This field is of high general interest for the broader scientific community. My expertise is in the field of membraneless organelles.

Review #3 -

Evidence, reproducibility and clarity (Required)

The authors examine the important and challenging question in current biology, the role of RNA in the structural maintenance of nuclear and cytoplasmic membrane-less organelles including stress granules, processing bodies, nucleolus, Cajal bodies, and nuclear speckles. Furthermore, the authors explored super-enhancer complexes involved in the regulation of gene expression. The authors used RNase L, an interferon-induced ribonuclease which, upon activation in the cytoplasm or targeted to the nucleus, degrades all RNAs within the cell. Then they took the quantitative approach to analyze the effect of RNA degradation on disassembly or reorganization of membrane-less organelles. Interestingly, the authors observed that RNAs present within nuclear organelles are susceptible to RNase IL degradation leading to their disassembly. In contrast, super-enhancer-containing eRNAs are largely unaffected.

Major concerns

Many studied organelles are challenging to see in many of the figures. Thus this reviewer encourages the authors to present clearer insets at higher magnification to illustrate what is being quantified, and then show that quantification in the central figure next to the immunofluorescent images.

The amount of specific RNAs degradation after induction of RNase L for several assemblies should be analyzed by qRT-PCR and quantified. This will justify observations provided by microscopy on an individual cell basis.
The main issue regards the connection between RNA and its role in the formation and structural integrity of nuclear organelles. There is consensus that these nuclear assemblies are built on specific nascent transcripts which act as a nucleation scaffold. If specific RNA synthesis is impaired, these assemblies collapse. The authors should discuss it. It would be relevant to mention two experimental works on this topics, DOI: 10.1038/ncb2140 and DOI: 10.1038/ncb2157
The study is limited to observed macroscopical changes in the appearances of assemblies. The authors must dig deeper and provide more conclusive results by several colocalizing components of these assemblies. It has been documented that the visualization of a selective marker for a specific assembly is not enough to prove its functionality/dysfunctionality but also the level of its disassembly. For example, in Figure 4A the authors should more convincible visualize nascent 47/45S pre-rRNA transcript to demonstrate that the nucleolus is built on ongoing pre-rRNA synthesis reflected by the tripartite nucleolar substructures. The loss of the GC component after rRNA depletion should be better presented with NPM1 colocalization.

In Figure 4C, D the authors used the term "coilin assemblies". That's confusing for a reader. The Cajal body after activation of RNase L likely undergoes the structural rearrangement which cannot be justified only by the presence of rearranged coilin foci. The authors should colocalize them with at least one or two functional markers.

Enhancer RNAs likely play the role in gene control rather than as a nucleation element to build nuclear assemblies. This should be discussed in the explanation of observed differences between MED1 foci and other assemblies.

3. Significance:

Significance (Required)

Understanding changes in the nuclear and cellular organization that accompany and drive changes in the formation and maintenance of cellular structures is an essential and not well-understood topic. Thus, this manuscript is relevant. However, the presented data in this paper are based on a limited approach, and particularly their interpretation and presentation could be substantially improved. Consequently, the conclusions are not convincingly supported by published data. However, some open questions need to be addressed. Specific criticisms are outlined above.

Manuscript number: EMBOJ-2021-110137 [RC-2021-01011]
Current title: **RNA is required for the integrity of multiple nuclear and cytoplasmic membrane-less RNP granules.**

Corresponding author(s): Roy Parker

Reviewer #1 (Evidence, reproducibility and clarity (Required)):

In the paper entitled. "RNA is required for the maintenance of multiple cytoplasmic and nuclear membrane-less organelles" Decker et al set out to test the rolw of RNA in maintaining the integrity of a variety of biomolecular condensates. To do this, they assess how multiple different assemblies in the cytoplasm and nucleus retain their structural integrity following RNAseL activation. They identified many condensates which are solubilized and have protein components redistributed following RNAse L activation and presumably subsequent RNA digestion. These experiments largely concur with previous findings from RNAseA treatment. The implication is that RNA rather than protein is the essential organizing component for most tested condensates. The manuscript is well written, and the data are convincing. It is my judgement that this is worthy of publication following a few additional experiments/clarifications.

1. The authors identify condensates which are sensitive to or refractory to RNAaseL. It would be good if the authors more conclusively eliminate the possibility that remaining condensates contain specific residual antiviral RNAs and this is the reason why these condensates remain intact. Are any of these condensates enriched in anti-viral RNAs like IFNbeta following polyIC treatment by FISH, for example (PMID: 31494035)?

In this comment, the reviewer is asking us to determine if condensates refractory to RNAse L (which in the original version were TIS granules), are refractory to RNase L because the contain some residual antiviral mRNAs. Since we posted our manuscript to bioRxiv we have discovered that the mesh-like assemblies we detect with the antibody used in the original paper describing TIS granules (Ma and Mayr 2018 doi.org/10.1016/j.cell.2018.10.007) are mitochondria. This is based on personal communications from Christine Mayr (the discoverer of TIS granules) and our observation that the mesh-like structures the antibody recognizes in A549 cells co-localize with a fluorescent mitochondrial stain (mitotracker Deep Red). The majority of the experiments describing TIS granules by Ma and Mayr used overexpressed fluorescent tagged TIS11B protein. Since we are unable to detect endogenous TIS granules in A549 cells, we have revised the manuscript to remove all references to TIS granules. This does not alter the main points of the manuscript.

2. Is there a particular protein feature, charge, IDR-types etc. which is common to solubilized versus not solubilized groups? What about dissolved and novel formed assemblies? A simple table comparing protein features in the three groups would suffice, with particular emphasis on RNA binding domains PMID: 32243832 and intrinsic disordered regions PMID: 24773235.

We have added to the discussion text describing the general principles of why a given assembly would be affected by RNA degradation or not and possible explanations why condensates might

form novel assemblies rather than solubilize in response to loss of RNA. For those condensates that tended to form novel assemblies we have discussed protein features that could contribute toward their formation.

3. Demonstrate that the RNAseL treatment is reversible (i.e. withdraw polyIC, particularly for a protein that ends up in a novel assembly) or remove the word maintenance from the title.

We agree with the reviewer that it would be an interesting experiment to examine the recovery of condensates after their assembly is disrupted by RNA degradation by RNase L. Unfortunately, the activation of RNase L cannot be reversed by simply washing out polyIC since a signaling molecule oligo2-5'A is made that activates RNase L. Since we cannot perform this experiment, we have revised the title as suggested by the reviewer.

4. Control for RNA-dependence of the activity. Try to dissolve a non-RNA dependent/enriched condensate with RNAseL. SPOP mutations (PMID: 30244836) might be interesting as both SPOP and RNAseL loss of function mutations (PMID: 11799394) are associated with prostate cancer.

The reviewer suggested that we examine the effect of RNase L activation on non-RNA dependent assemblies to confirm that they are not affected by the loss of RNA. To address this issue, we examined if RNase L activation affected the microtubule and intermediate filament networks in the cytosol, as well as the nuclear lamin and PML bodies in the nucleus. We observed no changes to these structures when RNase L was activated. This is consistent with the effects of RNase L on assemblies being due to the degradation of their encapsulated RNAs.

5. A caveat is that certain regions of condensates enriched in RNA may not be accessible to RNAseL protein. A way to address this might be to attempt to directly target the enzyme to a compartment that is deemed refractory to the activity (and inferred to not require RNA) via an inducible systsem (ie FKBP12/FK506)

The reviewer suggests we target RNase L to an RNP granule using additional protein-protein interactions to determine if the absence of an effect is due to the exclusion of RNase L. This is a good experiment and in the previous draft of the manuscript (Figure S1), we had described targeting RNase L to P-bodies through an RNase L-Dcp1 fusion protein to determine if that affected the number/size of P-bodies in response to RNase L activation. We did not observe an effect of reduction in P-bodies through the targeting of RNase L. This data was presented in a supplemental figure, and therefore might have been missed by the reviewer. We revised the manuscript to make it clearer that inaccessibility of RNA within P-bodies is likely not to be the reason that P-bodies are not disrupted when RNase L is activated.

6. Overall, this paper would be greatly enhanced by including a more extensive discussion on the basic biological implications for these findings. Why are some condensates RNA dependent? What function(s) are common to these condensates? How does disruption of this lead to disease?

We have revised the manuscript to discuss more extensively the implications of our findings.

Reviewer #1 (Significance (Required)):

This work addresses the neglected role of RNA in structuring condensates throughout the cell. Despite the prevalence of RNA in many condensates and the enrichment of RNA-binding proteins in condensates, there is still a highly limited understanding of the structural roles RNA plays in their assembly s most work has been protein/IDR-centric. This work seeks to systematically assess the RNA-dependence of the assemblies.

Reviewer #2 (Evidence, reproducibility and clarity (Required)):

The manuscript by Decker et al. "RNA is required for the maintenance of multiple cytoplasmic and nuclear membrane-less organelles" investigates the structural role of RNA in membraneless organelles. The authors show that degradation of RNA in transient or constitutive membraneless organelles results in the altered formation and structure of many but not all organelles studied. The main assay is the activation of RNAseL activity by dsRNA which then destroys mRNA in the cell. The collected data leads the authors to highlight the possible roles of RNA in membraneless organelle formation and categorize the organelles: some relying more on the RNA-RNA interactions while others on protein-RNA or protein-protein interactions. The manuscript is well written and the data is sound.

Major comments:

The authors study the maintenance of organelles by RNA. For the transient ones, like stress granules (SG), it would be very interesting to see the formation/clearance kinetics with and without RNA. Also maybe using something other than dsRNA to trigger the formation. The idea being - if RNA is needed for SG maintenance, then the clearance kinetics with RNA would differ from that of the depleted RNA.

In this comment, the reviewer is asking if RNA degradation can alter the disassembly rates of stress granules. We have previously described that activation of RNase L results in the disassembly of preformed stress granules and prevents the formation of stress granules in response to sodium arsenite (Burke et al. 2020 doi: 10.1074/jbc.RA119.011638). We revised the manuscript to emphasize this prior observation.

The experiments were done in cells. It is known that core components of the organelles can form granule like structures in vitro without RNA. If it is possible to show that RNA presence improves the integrity in vitro, that would support the authors claim. For example studying SG maintenance with and without RNAseL using the previously developed SG extraction protocol.

Consistent with the reviewer's suggestion, the analysis of granule-like structures in vitro supports the idea that RNA is important for their integrity. We have revised the manuscript to discuss these prior, relevant observations. The stress granule extraction protocol enriches for substructures of stress granules which are stable when treated with RNase (Jain et al 2016 doi: 10.1016/j.cell.2015.12.038) but this is due to the RNA in these substructures being inaccessible to RNases once cells are lysed (Van Treeck et al., 2018 doi:10.1016/j.cell.2018.07.023). The importance of RNA to stress granule assembly is supported by recent studies in which stress granule-like structures are assembled from cell lysates/cell fractions in which the formation of these structures is induced by the addition of RNA (Begovich and Wilhelm 2020 doi: 10.1016/j.molcel/2020.07/017) and inhibited by RNase treatment of lysates before assemblies are induced (Freibaum et al 2021 doi: 10.1083/jcb.202009079).

Minor comments:

In the Figure 1a, it is not clear if the smaller granules are different from SGs as mentioned in the text, maybe using additional markers can make it clearer.

We have previously described the characterization of these smaller granules (Burke et al. 2020 doi: 10.1074/jbc.RA119.011638). We revised the manuscript to clarify that these smaller granules are distinct from stress granules and have been previously characterized.

Figure 3 and 4 requires quantification. Figure 3 and 4 refer to current Figure 2 and 3 in the revised manuscript. We revised both the figures to include quantification.

Reviewer #2 (Significance (Required)):

This is a solid paper that advances our understanding of membraneless organelle formation and dynamics. This field is of high general interest for the broader scientific community. My expertise is in the field of membraneless organelles.

Reviewer #3 (Evidence, reproducibility and clarity (Required)):

The authors examine the important and challenging question in current biology, the role of RNA in the structural maintenance of nuclear and cytoplasmic membrane-less organelles including stress granules, processing bodies, nucleolus, Cajal bodies, and nuclear speckles. Furthermore, the authors explored super-enhancer complexes involved in the regulation of gene expression. The authors used RNase L, an interferon-induced ribonuclease which, upon activation in the cytoplasm or targeted to the nucleus, degrades all RNAs within the cell. Then they took the quantitative approach to analyze the effect of RNA degradation on disassembly or reorganization of membrane-less organelles. Interestingly, the authors observed that RNAs present within nuclear organelles are susceptible to RNase IL degradation leading to their disassembly. In contrast, super-enhancer-containing eRNAs

are largely unaffected.

Major concerns

Many studied organelles are challenging to see in many of the figures. Thus this reviewer encourages the authors to present clearer insets at higher magnification to illustrate what is being quantified, and then show that quantification in the central figure next to the immunofluorescent images.

As suggested by the reviewer, we revised the figures to present the granules and the effect of RNA loss on their assembly more clearly. We have also quantified the effects of RNA loss on the granules in figures.

The amount of specific RNAs degradation after induction of RNase L for several assemblies should be analyzed by qRT-PCR and quantified. This will justify observations provided by microscopy on an individual cell basis.

We believe that examination of the effects of RNase L on RNAs is best done at the single cell level by smFISH. This is because not all cells activate RNase L in response to transfection with poly I:C (Burke et al, 2019 doi: 10.1016/j.molcel.2019.07.029) and Fig2D, and therefore qRT-PCR of the bulk population of transfected cells will not accurately reflect changes in RNA levels in the cells that do activate RNase L. Microscopy allows us to discern which cells contain active RNase L by monitoring the degradation of polyA+ RNA by oligod(T) FISH and/or RNAs that are present in particular granules and then examine in those specific responding cells the effect of RNA loss on membraneless organelles.

The main issue regards the connection between RNA and its role in the formation and structural integrity of nuclear organelles. There is consensus that these nuclear assemblies are built on specific nascent transcripts which act as a nucleation scaffold. If specific RNA synthesis is impaired, these assemblies collapse. The authors should discuss it. It would be relevant to mention two experimental works on this topics, DOI: 10.1038/ncb2140 and DOI: 10.1038/ncb2157

We revised the manuscript to discuss previous work arguing that nuclear RNP granules organelles are assembled on specific transcripts and/or at sites of nascent transcription.

The study is limited to observed macroscopical changes in the appearances of assemblies. The authors must dig deeper and provide more conclusive results by several colocalizing components of these assemblies. It has been documented that the visualization of a selective marker for a specific assembly is not enough to prove its functionality/dysfunctionality but also the level of its disassembly. For example, in Figure 4A the authors should more convincible visualize nascent 47/45S pre-rRNA transcript to demonstrate that the nucleolus is built on ongoing pre-rRNA synthesis

reflected by the tripartite nucleolar substructures. The loss of the GC component after rRNA depletion should be better presented with NPM1 colocalization.

As suggested by the reviewer, we examined additional protein components for each assembly. We also examined how RNase L activation in the nucleus affects the levels of 47/45S pre-rRNA, and the relationship of the loss of such pre-rRNA and nucleolar assembly.

In Figure 4C, D the authors used the term "coilin assemblies". That's confusing for a reader. The Cajal body after activation of RNase L likely undergoes the structural rearrangement which cannot be justified only by the presence of rearranged coilin foci. The authors should colocalize them with at least one or two functional markers.

As suggested by the reviewer, we examined how the SMN1 protein, which associates with Cajal bodies, is affected by nuclear RNA degradation. We observed that SMN1 is distributed in the nucleoplasm and does not co-localize with the residual coilin RNA-independent assemblies that can form. This argues that SMN1 association with coilin is disrupted when RNA is depleted.

Enhancer RNAs likely play the role in gene control rather than as a nucleation element to build nuclear assemblies. This should be discussed in the explanation of observed differences between MED1 foci and other assemblies.

We revised the manuscript to discuss more thoroughly why transcriptional condensates are not affected by degradation of nuclear RNA.

Reviewer #3 (Significance (Required)):

Understanding changes in the nuclear and cellular organization that accompany and drive changes in the formation and maintenance of cellular structures is an essential and not well-understood topic. Thus, this manuscript is relevant. However, the presented data in this paper are based on a limited approach, and particularly their interpretation and presentation could be substantially improved. Consequently, the conclusions are not convincingly supported by published data. However, some open questions need to be addressed. Specific criticisms are outlined above.

1st Revision - Editorial Decision **21st Feb 2022**

Thank you again for submitting your revised manuscript. We have received comments from the initial three referees (please see below) and I am pleased to say that they now support publication. Referee #2 has some remaining comments that can be addressed in the final revised version of the manuscript. In addition, in this version, I would ask you to please resolve a number of editorial issues that are listed in detail below. Please feel free to contact me if you have further questions regarding the revision or any of the specific points listed below. Thank you again for giving us the chance to consider your manuscript for The EMBO Journal.

Referee #1:

The authors have nicely addressed our concerns and we think this is a valuable body of data to publish.

Referee #2:

This is a significantly improved manuscript with an amount of compelling data that support the idea that specific RNA molecules are required for the functional integrity of RNP granules in the nucleus and the cytoplasm. In my opinion, the manuscript is suitable for publication in EMBO J.

Additional points

Page 11-13. The authors should better justify the observed data concerning the structural integrity of Cajal bodies in response to nuclear RNA degradation. It has been established that the nucleation and formation of a Cajal body are triggered by the large ubiquitously expressed gene arrays, such as the spliceosomal U1 and U2 snRNA arrays. Subsequently, other highly expressed U snRNA genes, which are positioned at the periphery of their host chromosome territory associate with the growing Cajal body (DOI: 10.1038/ncomms10966 | www.nature.com/naturecommunication). Thus, the highly abundant nascent unprocessed U snRNA transcripts act as scaffolds for a Cajal body formation (https://doi.org/10.1002/bies.201600144). In contrast, there is no indication that the Cajal body contains any polyadenylated nuclear RNAs. However, published data indicate that the Cajal body is always formed in the closest proximity of nuclear speckles where pre-mRNA splicing occurs at their periphery. Therefore, the observed data that degradation of nuclear poly(A) RNA by activated RNase L likely reflects the impaired dynamic turnover of the spliceosomal snRNPs involved in RNA splicing. Consequently, the reduced levels of splicing events lead to the decreased fluxes of snRNP molecules to the Cajal body and are followed by their dynamic functional disintegration (DOI: 10.1038/ncomms10966 | www.nature.com/naturecommunication).

Referee #3:

The authors have done a great job addressing the concerns of the reviewers. I support publication.

Response to Reviews:

Referee #1:

The authors have nicely addressed our concerns and we think this is a valuable body of data to publish.

Terrific!

Referee #2:

This is a significantly improved manuscript with an amount of compelling data that support the idea that specific RNA molecules are required for the functional integrity of RNP granules in the nucleus and the cytoplasm. In my opinion, the manuscript is suitable for publication in EMBOJ.

Terrific!

Additional points

Page 11-13. The authors should better justify the observed data concerning the structural integrity of Cajal bodies in response to nuclear RNA degradation. It has been established that the nucleation and formation of a Cajal body are triggered by the large ubiquitously expressed gene arrays, such as the spliceosomal U1 and U2 snRNA arrays. Subsequently, other highly expressed U snRNA genes, which are positioned at the periphery of their host chromosome territory associate with the growing Cajal body (DOI: 10.1038/ncomms10966 | www.nature.com/naturecommunication). Thus, the highly abundant nascent unprocessed U snRNA transcripts act as scaffolds for a Cajal body formation (https://doi.org/10.1002/bies.201600144). In contrast, there is no indication that the Cajal body contains any polyadenylated nuclear RNAs. However, published data indicate that the Cajal body is always formed in the closest proximity of nuclear speckles where pre-mRNA splicing occurs at their periphery. Therefore, the observed data that degradation of nuclear poly(A) RNA by activated RNase L likely reflects the impaired dynamic turnover of the spliceosomal snRNPs involved in RNA splicing. Consequently, the reduced levels of splicing events lead to the decreased fluxes of snRNP molecules to the Cajal body and are followed by their dynamic functional disintegration (DOI: 10.1038/ncomms10966 | www.nature.com/naturecommunication).

In response to the Referee #2's comment regarding the response of Cajal bodies to nuclear RNA degradation we have revised the manuscript to make it clear to readers that the formation of Cajal bodies can be nucleated by the transcription of arrays of snRNA genes so one might expect that their de novo formation would be defective in response to nuclear degradation. We have also clarified that we are using the loss of polyadenylated RNA as a marker for nuclei in which RNA has been degraded. We did not

mean to imply that Cajal bodies contain polyadenylated RNA. The reviewer suggests that degradation of nuclear polyadenylated RNA reduces the level of splicing events that would then decrease the flux of snRNP molecules to Cajal bodies and lead to their disintegration. A more parsimonious explanation is that active RNase L in the nucleus degrades snRNAs, as well as other RNAs that move in and out of Cajal bodies, and this leads to the changes in the Cajal body morphology that we observe. These clarifications are tracked in the revised version of the manuscript (Introduction page 3, Results pages 10-11).

Referee #3:

The authors have done a great job addressing the concerns of the reviewers. I support publication.

Terrific!

ACCEPTED **14th Mar 2022**

Thank you again for submitting the final revised version of your manuscript. I am pleased to inform you that we have now accepted it for publication in The EMBO Journal.

EMBO PRESS

YOU MUST COMPLETE ALL CELLS WITH A PINK BACKGROUND 🡻

PLEASE NOTE THAT THIS CHECKLIST WILL BE PUBLISHED ALONGSIDE YOUR PAPER

Corresponding Author Name: Roy Parker
Journal Submitted to: EMBO Journal
Manuscript Number: EMBOJ-2021-110137

Reporting Checklist For Life Sciences Articles (Rev. June 2017)

This checklist is used to ensure good reporting standards and to improve the reproducibility of published results. These guidelines are consistent with the Principles and Guidelines for Reporting Preclinical Research issued by the NIH in 2014. Please follow the journal's authorship guidelines in preparing your manuscript.

A- Figures
1. Data
The data shown in figures should satisfy the following conditions:

- the data were obtained and processed according to the field's best practice and are presented to reflect the results of the experiments in an accurate and unbiased manner.
- figure panels include only data points, measurements or observations that can be compared to each other in a scientifically meaningful way.
- graphs include clearly labeled error bars for independent experiments and sample sizes. Unless justified, error bars should not be shown for technical replicates.
- if n< 5, the individual data points from each experiment should be plotted and any statistical test employed should be justified
- Source Data should be included to report the data underlying graphs. Please follow the guidelines set out in the author ship guidelines on Data Presentation.

2. Captions

Each figure caption should contain the following information, for each panel where they are relevant:

- a specification of the experimental system investigated (eg cell line, species name).
- the assay(s) and method(s) used to carry out the reported observations and measurements
- an explicit mention of the biological and chemical entity(ies) that are being measured.
- an explicit mention of the biological and chemical entity(ies) that are altered/varied/perturbed in a controlled manner.

- the exact sample size (n) for each experimental group/condition, given as a number, not a range;
- a description of the sample collection allowing the reader to understand whether the samples represent technical or biological replicates (including how many animals, litters, cultures, etc.).
- a statement of how many times the experiment shown was independently replicated in the laboratory.
- definitions of statistical methods and measures:
  - common tests, such as t-test (please specify whether paired vs. unpaired), simple χ2 tests, Wilcoxon and Mann-Whitney tests, can be unambiguously identified by name only, but more complex techniques should be described in the methods section;
  - are tests one-sided or two-sided?
  - are there adjustments for multiple comparisons?
  - exact statistical test results, e.g., P values = x but not P values < x;
  - definition of 'center values' as median or average;
  - definition of error bars as s.d. or s.e.m.

Any descriptions too long for the figure legend should be included in the methods section and/or with the source data.

In the pink boxes below, please ensure that the answers to the following questions are reported in the manuscript itself. Every question should be answered. If the question is not relevant to your research, please write NA (non applicable). We encourage you to include a specific subsection in the methods section for statistics, reagents, animal models and human subjects.

USEFUL LINKS FOR COMPLETING THIS FORM

http://www.antibodypedia.com
http://1degreebio.org
http://www.equator-network.org/reporting-guidelines/improving-bioscience-research-repor
http://grants.nih.gov/grants/olaw/olaw.htm
http://www.mrc.ac.uk/Ourresearch/Ethicsresearchguidance/Useofanimals/index.htm
http://ClinicalTrials.gov
http://www.consort-statement.org
http://www.consort-statement.org/checklists/view/32-consort/66-title
http://www.equator-network.org/reporting-guidelines/reporting-recommendations-for-tumo
http://datadryad.org
http://figshare.com
http://www.ncbi.nlm.nih.gov/gap
http://www.ebi.ac.uk/ega
http://biomodels.net/
http://biomodels.net/miriam/
http://jjj.biochem.sun.ac.za
https://osp.od.nih.gov/biosafety-biosecurity-and-emerging-biotechnology/
http://www.selectagents.gov/

B- Statistics and general methods

Please fill out these boxes 🡻 (Do not worry if you cannot see all your text once you press return)

1.a. How was the sample size chosen to ensure adequate power to detect a pre-specified effect size?	Sample size was determined by practical limitations on imaging and analysis of images using Imaris 3D imaging analysis. Biological replicates were analyzed to yield more accurate and reliable summary statistics.
1.b. For animal studies, include a statement about sample size estimate even if no statistical methods were used.	not applicable
2. Describe inclusion/exclusion criteria if samples or animals were excluded from the analysis. Were the criteria pre-established?	samples were not excluded
3. Were any steps taken to minimize the effects of subjective bias when allocating animals/samples to treatment (e.g. randomization procedure)? If yes, please describe.	not applicable
For animal studies, include a statement about randomization even if no randomization was used.	not applicable
4.a. Were any steps taken to minimize the effects of subjective bias during group allocation or/and when assessing results (e.g. blinding of the investigator)? If yes please describe.	no
4.b. For animal studies, include a statement about blinding even if no blinding was done	not applicable
5. For every figure, are statistical tests justified as appropriate?	yes
Do the data meet the assumptions of the tests (e.g., normal distribution)? Describe any methods used to assess it.	Normalcy was assessed using suite of tests provided in Prism 9.0. Non parametric tests were used when data did not fit normal distribution.
Is there an estimate of variation within each group of data?	yes

Is the variance similar between the groups that are being statistically compared?	yes

C- Reagents

6. To show that antibodies were profiled for use in the system under study (assay and species), provide a citation, catalog number and/or clone number, supplementary information or reference to an antibody validation profile. e.g., Antibodypedia (see link list at top right), 1DegreeBio (see link list at top right).	catalog numbers provided for all antibodies
7. Identify the source of cell lines and report if they were recently authenticated (e.g., by STR profiling) and tested for mycoplasma contamination.	yes

* for all hyperlinks, please see the table at the top right of the document

D- Animal Models

8. Report species, strain, gender, age of animals and genetic modification status where applicable. Please detail housing and husbandry conditions and the source of animals.	not applicable
9. For experiments involving live vertebrates, include a statement of compliance with ethical regulations and identify the committee(s) approving the experiments.	not applicable
10. We recommend consulting the ARRIVE guidelines (see link list at top right) (PLoS Biol. 8(6), e1000412, 2010) to ensure that other relevant aspects of animal studies are adequately reported. See author guidelines, under 'Reporting Guidelines'. See also: NIH (see link list at top right) and MRC (see link list at top right) recommendations. Please confirm compliance.	not applicable

E- Human Subjects

11. Identify the committee(s) approving the study protocol.	not applicable
12. Include a statement confirming that informed consent was obtained from all subjects and that the experiments conformed to the principles set out in the WMA Declaration of Helsinki and the Department of Health and Human Services Belmont Report.	not applicable
13. For publication of patient photos, include a statement confirming that consent to publish was obtained.	not applicable
14. Report any restrictions on the availability (and/or on the use) of human data or samples.	not applicable
15. Report the clinical trial registration number (at ClinicalTrials.gov or equivalent), where applicable.	not applicable
16. For phase II and III randomized controlled trials, please refer to the CONSORT flow diagram (see link list at top right) and submit the CONSORT checklist (see link list at top right) with your submission. See author guidelines, under 'Reporting Guidelines'. Please confirm you have submitted this list.	not applicable
17. For tumor marker prognostic studies, we recommend that you follow the REMARK reporting guidelines (see link list at top right). See author guidelines, under 'Reporting Guidelines'. Please confirm you have followed these guidelines.	not applicable

F- Data Accessibility

18: Provide a "Data Availability" section at the end of the Materials & Methods, listing the accession codes for data generated in this study and deposited in a public database (e.g. RNA-Seq data: Gene Expression Omnibus GSE39462, Proteomics data: PRIDE PXD000208 etc.) Please refer to our author guidelines for 'Data Deposition'.<br><br>Data deposition in a public repository is mandatory for:<br>a. Protein, DNA and RNA sequences<br>b. Macromolecular structures<br>c. Crystallographic data for small molecules<br>d. Functional genomics data<br>e. Proteomics and molecular interactions	Microscopy Image Files: BioStudies S-BIAD359
19. Deposition is strongly recommended for any datasets that are central and integral to the study; please consider the journal's data policy. If no structured public repository exists for a given data type, we encourage the provision of datasets in the manuscript as a Supplementary Document (see author guidelines under 'Expanded View' or in unstructured repositories such as Dryad (see link list at top right) or Figshare (see link list at top right).	Microscopy Image Files: BioStudies S-BIAD359
20. Access to human clinical and genomic datasets should be provided with as few restrictions as possible while respecting ethical obligations to the patients and relevant medical and legal issues. If practically possible and compatible with the individual consent agreement used in the study, such data should be deposited in one of the major public access-controlled repositories such as dbGAP (see link list at top right) or EGA (see link list at top right).	not applicable
21. Computational models that are central and integral to a study should be shared without restrictions and provided in a machine-readable form. The relevant accession numbers or links should be provided. When possible, standardized format (SBML, CellML) should be used instead of scripts (e.g. MATLAB). Authors are strongly encouraged to follow the MIRIAM guidelines (see link list at top right) and deposit their model in a public database such as Biomodels (see link list at top right) or JWS Online (see link list at top right). If computer source code is provided with the paper, it should be deposited in a public repository or included in supplementary information.	not applicable

G- Dual use research of concern

22. Could your study fall under dual use research restrictions? Please check biosecurity documents (see link list at top right) and list of select agents and toxins (APHIS/CDC) (see link list at top right). According to our biosecurity guidelines, provide a statement only if it could.	no
